# Sarcolipin alters SERCA1a interdomain communication by impairing binding of both calcium and ATP

**DOI:** 10.1038/s41598-021-81061-6

**Published:** 2021-01-15

**Authors:** Cédric Montigny, Dong Liang Huang, Veronica Beswick, Thomas Barbot, Christine Jaxel, Marc le Maire, Ji-Shen Zheng, Nadège Jamin

**Affiliations:** 1 CEA, CNRS, Institute for Integrative Biology of the Cell (I2BC), Université Paris-Saclay, 91198 Gif-sur-Yvette, France; 2 Department of Physics, Evry-Val-d’Essonne University, 91025 Evry, France; 3 Hefei National Laboratory for Physical Sciences at the Microscale, School of Life Sciences, University of Science and Technology of China, Hefei, 230027 China

**Keywords:** Enzyme mechanisms, Biochemistry, Membrane proteins

## Abstract

Sarcolipin (SLN), a single-spanning membrane protein, is a regulator of the sarco-endoplasmic reticulum Ca^2+^-ATPase (SERCA1a). Chemically synthesized SLN, palmitoylated or not (pSLN or SLN), and recombinant wild-type rabbit SERCA1a expressed in *S. cerevisiae* design experimental conditions that provide a deeper understanding of the functional role of SLN on the regulation of SERCA1a. Our data show that chemically synthesized SLN interacts with recombinant SERCA1a, with calcium-deprived E2 state as well as with calcium-bound E1 state. This interaction hampers the binding of calcium in agreement with published data. Unexpectedly, SLN has also an allosteric effect on SERCA1a transport activity by impairing the binding of ATP. Our results reveal that SLN significantly slows down the E2 to Ca_2_.E1 transition of SERCA1a while it affects neither phosphorylation nor dephosphorylation. Comparison with chemically synthesized SLN deprived of acylation demonstrates that palmitoylation is not necessary for either inhibition or association with SERCA1a. However, it has a small but statistically significant effect on SERCA1a phosphorylation when various ratios of SLN-SERCA1a or pSLN-SERCA1a are tested.

## Introduction

**Figure 1:**
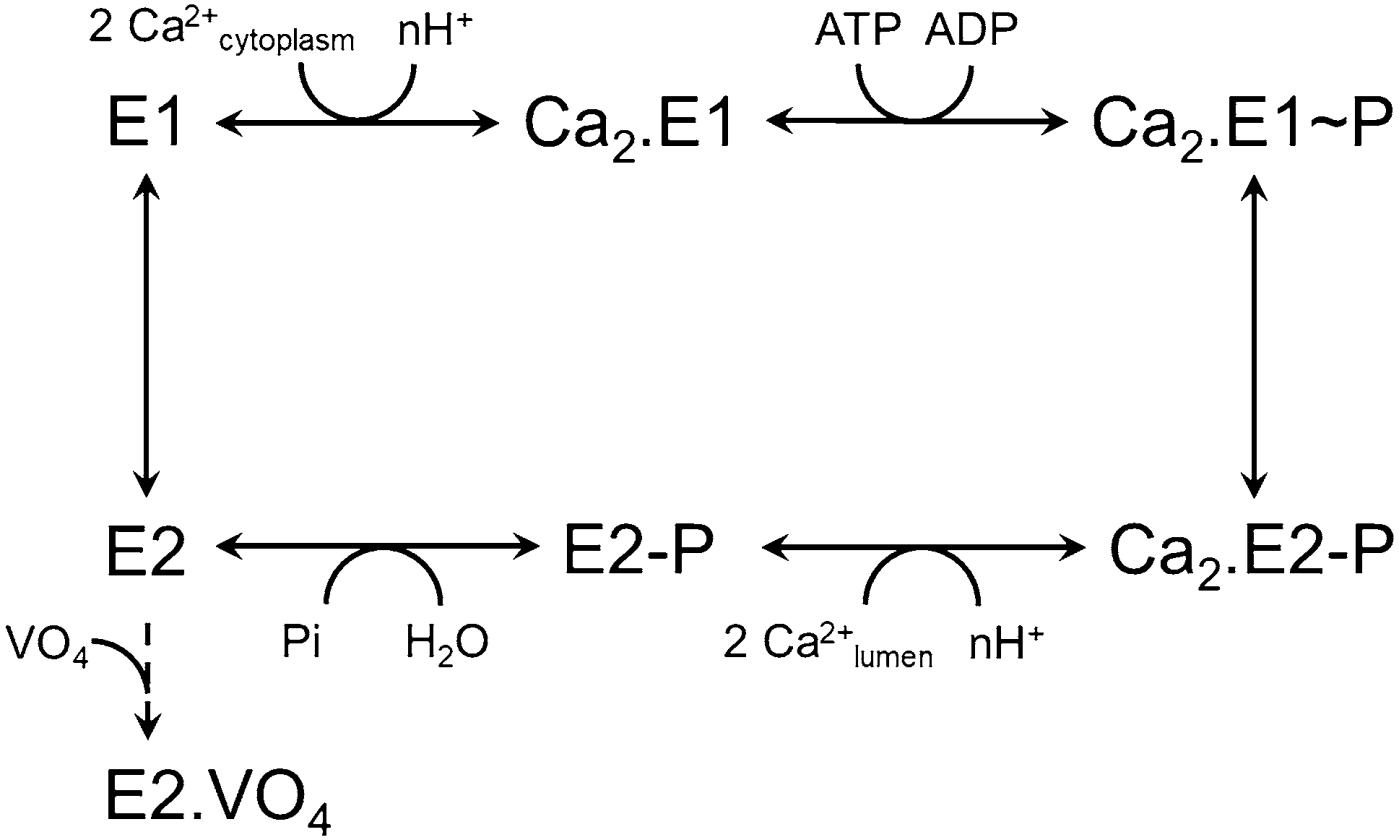
SERCA1a reaction cycle. Scheme shows the main steps for substrates binding and dissociation. At physiological pH, two cytoplasmic calcium ions bind to the “E1” high-affinity states. Binding of one Mg.ATP triggers calcium occlusion and allows autophosphorylation on the catalytic aspartate localized in P-domain (“Ca_2_.E1 ~ P” occluded state). The energy transferred from the nucleotide to the protein allows large conformational changes necessary for transport sites reorientation towards the lumen and then, for releasing of calcium ions against a concentration gradient. Protonation of the transport sites and autodephosphorylation of the Ca^2+^-free “E2-P” state enable cycling of the enzyme. Note that ATP can bind to most of these intermediates to act as a positive modulator and to accelerate transitions^1^. Orthovanadate (VO_4_) can act as an inhibitor after binding to the E2 ground state.

The sarco-endoplasmic reticulum Ca^2+^-ATPase (SERCA1a) is a 110 kDa integral membrane transporter and is one of the major actor of calcium homeostasis in fast-twitch muscle. SERCA1a uses ATP as an energy source to drive transport of calcium ions into the sarco-endoplasmic reticulum (SR) lumen to promote muscle relaxation. During catalysis, as described by the Post-Albers scheme^1^, SERCA1a switches between Ca^2+^-bound E1 states and Ca^2+^-free E2 states (Fig. 1).

Over the past 20 years, a number of high-resolution crystal structures of SERCA1a have been determined^1^. SERCA1a is composed of a 10 transmembrane spans membrane domain (M1 to M10), and of a large cytosolic headpiece comprising three domains: the nucleotide binding domain (N domain), the domain of phosphorylation (P domain) which possesses the conserved aspartate residue that is transiently phosphorylated during the transport cycle, and the actuator domain (domain A) which triggers dephosphorylation of the aspartate for cycling of the enzyme (Supplementary Fig. S1A). Among more than seventy structures, only three were resolved in the presence of a peptidic regulator i.e. phospholamban (PLB)^2^ or sarcolipin (SLN)^3,4^. PLB and SLN share the same binding groove within the Ca^2+^-ATPase, between transmembrane helices M2, M6 and M9. Interestingly, the same groove is occupied by lipids in some structures^5^. Whereas PLB is 52 amino-acid long, with a transmembrane helix and a long cytosolic extension that interacts with the N domain of SERCA1a^6^, SLN is only 31 amino-acid long and is composed of a transmembrane helix with very short cytosolic and luminal interfacial stretches^7^ (see Supplementary Fig. S2A). PLB and SLN act as inhibitors by slightly reducing affinity for calcium but their mechanism of inhibition of SERCA1a differs: the 20 residues long cytosolic domain of PLB is critical for inhibition of SERCA1a, while the short RSYQY luminal tail of SLN appears to be essential^8^. A few studies strongly suggest that PLB associates with SERCA1a only during the calcium binding step^9,10^ while SLN resides bound to the ATPase during the whole catalytic cycle^11^. However, the precise role of SLN at the different steps of the cycle remains unclear and need to be further experimentally investigated.

In addition, PLB and SLN undergo post-translational modifications. Residues in their N-terminus are target for kinases and phosphorylation affects association with the pump^12–14^. Consequently, phosphorylation of residues localized in PLB or SLN N-termini seems to be important for association to SERCA isoforms in the groove as illustrated by molecular models^13^. More recently, we reported that rabbit SLN is S-palmitoylated or S-oleoylated on cysteine while the human isoform is not because lacking a cysteine residue, having a phenylalanine residue instead^15^. The exact role of this acylation was not addressed even if it has been shown to be functionally relevant recently for other P-type ATPases regulatory subunits as PLB and phospholemman (PLM)^16–18^.

An analysis of all the published data on biochemical characterization of PLB or SLN highlights heterogeneity in the results. Whereas all experiments show that SLN induces a moderate decrease on the affinity for calcium, the reported impacts on the turn-over rate vary showing increasing or decreasing rates or even no effect: these variations are probably due to different experimental conditions (^13^ and Supplementary Table 1). PLB and SLN are transmembrane peptides mostly expressed in cardiac muscle and skeletal muscle respectively, and consequently interacting most probably with the SERCA2a and SERCA1a isoforms respectively. Although these two isoforms share a high amino acid sequence identity, the presence of tissue-specific ATPase and regulatory peptides most probably reveal fine-tuning of the ATPases activities that is not yet fully understood. Nevertheless, most of the studies were done using SERCA1a (from native sources or after heterologous expression) without taking into account the nature of the regulatory peptide (PLB or SLN), the animal species, or the presence of some endogenous regulatory peptide coming from sample preparation which can lead to a misestimate of the regulatory peptide to SERCA1a ratio (Supplementary table 1 and references herein). Whereas recombinant expression and purification of human SLN has been performed^19,20^, heterologous expression of rabbit SLN, an acylated peptide, is more problematic in particular because it is difficult to control the yield of acylation. Additionally, the hydrophobicity of SLN makes its purification from native sources such as SR very tricky^7^. Experiments performed using purified native SLN gave inconsistent results. While some groups reported an effect of co-reconstitution of native SLN with native SERCA iosforms either on the turn-over rate or the accumulation of calcium^11,21^, others did not observe such an effect though they used similar native sample^22^. As a matter of fact, we demonstrated by combining Size Exclusion Chromatography (SEC) and MALDI-TOF analysis of full-length proteins that mild detergent are unable to totally dissociate native rabbit SLN:SERCA1a complexes^15^: 20 to 30 percent of endogenous SLN remains bound to SERCA1a after SEC as estimated from SDS-PAGE after modification of tryptophan by haloalkanes. Therefore, preparation of purified SERCA1a from native source contains some endogenous SLN that should be taken into account in the stoichiometry (Supplementary table 1 and references herein). Later on, it was shown that co-expression of SERCA1a and SLN in mammalian cells can lead to a lowering of the affinity for calcium and also to an increase of the turn-over rate^23^. Therefore, in view of the discrepancies among the published data, measurements of kinetics data using a well-defined experimental set-up is essential for a deeper understanding of the regulatory role of SLN. In particular, in order to measure the phosphorylation and turn-over rates, a control of the amount of properly assembled SLN:SERCA1a complexes is mandatory. Although the reconstitution of SERCA1a alone or in the presence of PLB leads to about 80% of right side oriented proteins^24,25^, it has been shown that, for membrane proteins including SERCA1a, it can be particularly difficult to control the orientation after co-reconstitution (see Supplementary information and references herein). Thus, we chose to work with DDM or C_12_E_8_ solubilized proteins instead of reconstituted. This strategy was already successful for structural analysis by NMR of the SLN:SERCA1a complex in dodecylphosphocholine^26^. In addition, we and others have shown that DDM- or C_12_E_8_-solubilized native or recombinant S1a are stable for long period from a few hours to days depending on the ligands. This solubilized enzyme behaves as the native SERCA1a^27,28^ (see also Supplementary information and references herein).

In the present study, we used chemically synthesized S-palmitoylated and non-palmitoylated rabbit SLN (dubbed “pSLN” and “SLN”, respectively)^29^, as well as recombinant rabbit SERCA1a produced in the yeast *S. cerevisiae*^30^ to investigate the regulatory role of SLN on SERCA1a. Therefore, in our experimental set-up, the two proteins are (i) from the same species, i.e. rabbit, (ii) deprived from any endogenous regulatory peptide as no SERCA isoforms nor PLB nor SLN homologues have been detected in *S. cerevisiae*^31^. Moreover, the stoichiometry can be tightly controlled in our experiments. In addition, the role of the palmitoylation of SLN on the regulation of SERCA1a can be investigated. We show that our synthetic peptide is biologically active whether acylated or not, thus providing a deeper understanding of the functional role of SLN. In particular, we reveal that both pSLN and SLN slows down the E2 to Ca_2_.E1 transition of recombinant wild-type rabbit SERCA1a as demonstrated by using phosphorylation from radiolabelled ATP. We show that SLN has an allosteric effect on SERCA1a by impairing both the binding of calcium and the binding of ATP. Thus, our results demonstrate that even if SLN shares some properties with PLB in its mechanism of regulation of SERCA1a, it also presents important specificities.

## Results

### Chemical synthesis of Palm-SLN and SLN

**Figure 2:**
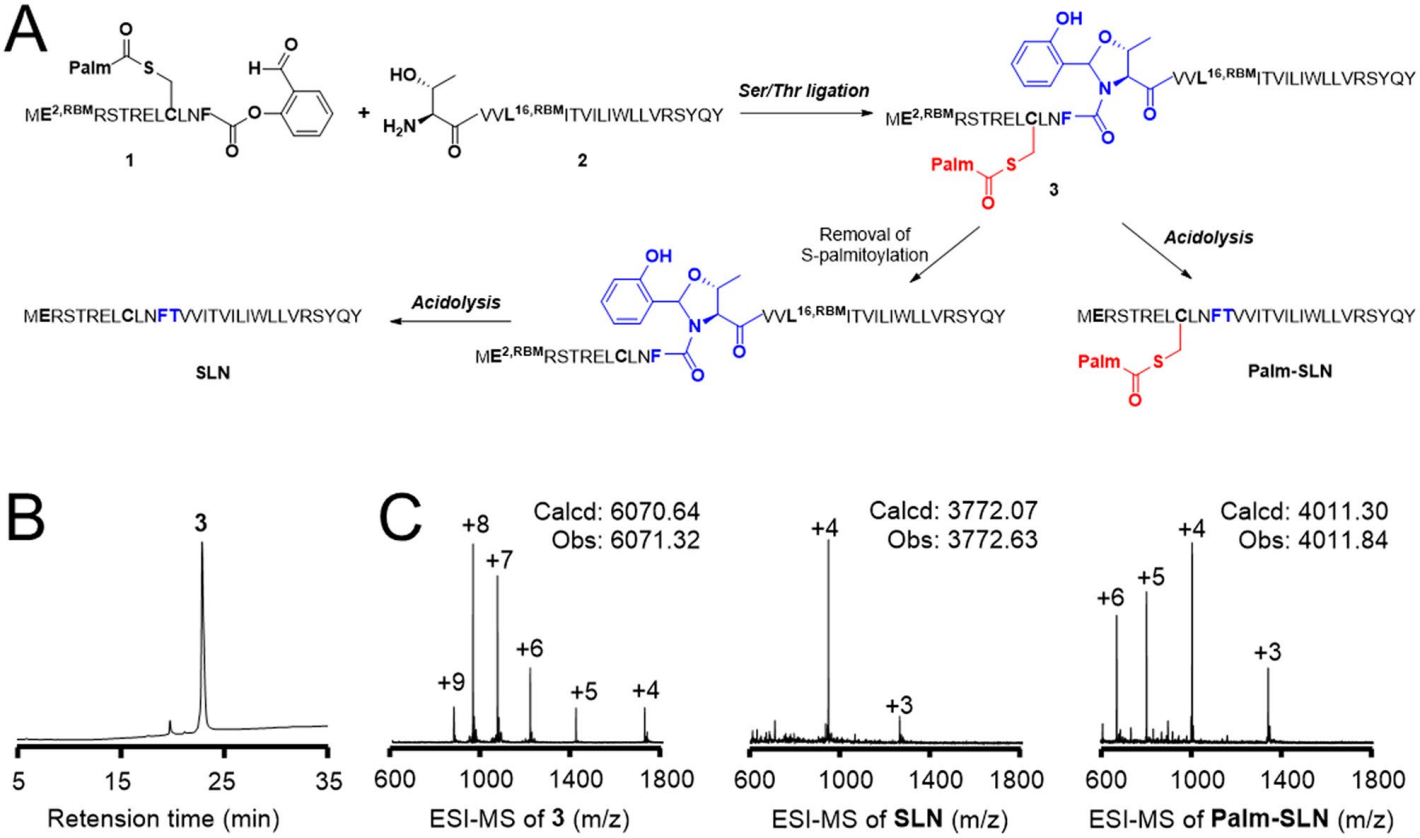
Chemical synthesis of pSLN and SLN by the RBM^GABA^-based STL method. (**A**) The synthetic route. (**B**) The RP-HPLC profile of purified 3. (**C**) ESI–MS analysis of 3, Palm-SLN and SLN.

We demonstrated that rabbit SLN is fully acylated (palmitoylated or oleoylated) in rabbit SR on its Cys9^15^. To investigate a possible role of palmitoylation on SERCA1a activity, pSLN and SLN were chemically synthesized by a Ser/Thr ligation (STL) method (Fig. 2a) following the protocol we designed recently^29^. In brief, taking benefit of the presence of Thr13, two peptides (peptides 1 and 2, Fig. 2a and Supplementary Fig. 3, upper and middle panels) were synthesized by the γ-aminobutyric acid- (GABA-) based removable backbone modification strategy. We then proceed to ligation between peptides 1 and 2 to afford the intermediate 3. This product was purified by reverse-phase high-performance liquid chromatography (RP-HPLC, Fig. 2b and Supplementary Fig. 3, lower panel) and it was verified by electrospray ionization mass spectrometry (ESI–MS, Fig. 2c, left panel). Afterwards, purified product 3 was dissolved in an acidic cocktail (0.1 M HCl and 1% tri-isopropylsilane (TIPS) in hexafluoroisopropanol (HFIP)) at room temperature to obtain the final product SLN. Yield of synthesis is about 53%. pSLN was also characterized by ESI–MS (Fig. 2c, right panel, and Supplementary Fig. 4). For the preparation of unacylated SLN, product 3 was treated with 5% hydrazine for 30 min to quantitatively remove the S-palmitoylate group. The RBM-modified SLN was then treated with the acidic cocktail to obtain the native SLN. Yield of synthesis for SLN is about 42%. SLN was characterized by ESI–MS (Fig. 2c, middle, and Supplementary Fig. 4).

### Sarcolipin interacts with Ca_2_.E1 and also with E2.Tg states

We have used limited proteolysis to follow SLN binding to SERCA1a under different conformations. Limited proteolysis using Proteinase K (PrtK) has been extensively used to discriminate E1-like states from E2-like states, and therefore to evidence overall conformational changes of either the cytoplasmic domain or the transmembrane domain, revealed by the binding of several ligands such as calcium, nucleotide or inhibitors^32,33^. Indeed, proteinase K cleavage is relatively unspecific for amino-acids and is, however, dependent on the accessibility of the amino-acyl chain. Proteinase K treatment in the presence of calcium to form a Ca_2_.E1 state leads to a major cleavage of SERCA1a at position T242-E243 that can be detected on SDS-PAGE as two major bands at 83 kDa and 28 kDa (dubbed as p83C and p28N respectively, see Supplementary Fig. 1B, lanes 1–3). In parallel, the presence of EGTA and thapsigargin (Tg) (to form an E2-like state) leads to another cleavage at position L119-K120, resulting in additional fragments of 95 kDa and 14 kDa (dubbed as p95C and p14N respectively, see Supplementary Fig. 1B, lane 4–6). Those different proteolysis profiles illustrate changes in the orientation of the A-domain and in the conformation of the M2-A and A-M3 linkers (Supplementary Fig. 1A).

**Figure 3:**
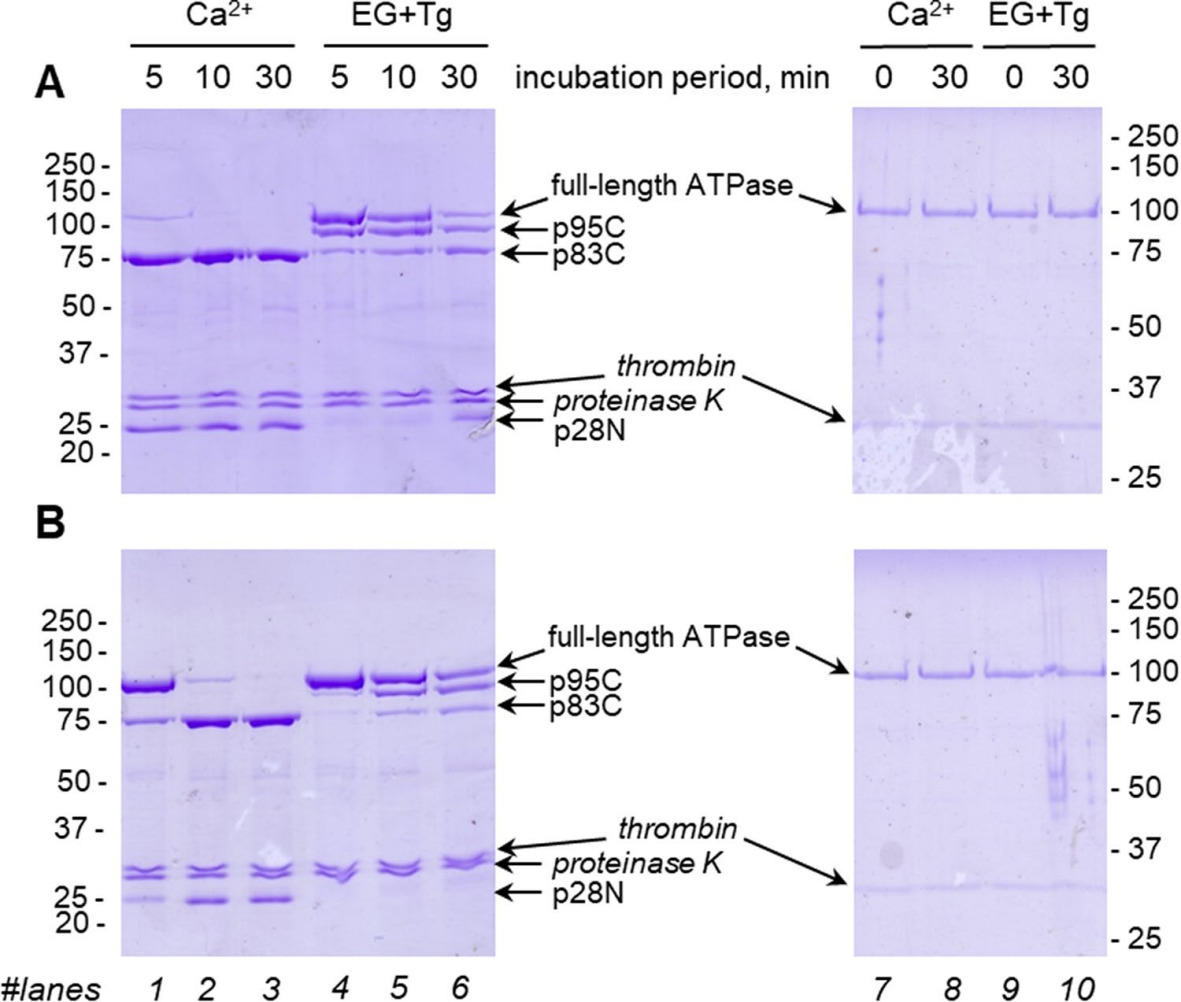
Effect of SLN during proteinase K treatment of recombinant purified SERCA1a (recS1a). (**A**) without SLN; (**B**) with palmitoylated SLN. Proteinase K proteolysis was performed in the presence of either 1 mM calcium (lanes 1–3) (cleavage at T242-E243) or EGTA and Thapsigargin (EG + Tg) (lanes 4–6) (cleavage at L119-K120) for 5 to 30 min before loading on SDS-PAGE as mentioned in the Methods section. Samples were also incubated for 30 min at 20 °C in absence of Proteinase K as standard, either in presence of 1 mM calcium (lanes 7–8) or EGTA and Tg (lanes 9–10), to assess the stability of the DDM-solubilized proteins. Thrombin, used for elution from the affinity chromatography resin, is present in the purified sample.

To date, proteolysis experiments have been done only on the native SERCA1a extracted from the rabbit skeletal muscle (dubbed “SR” below for sarco-endoplasmic reticulum). Since SERCA1a was embedded in its native membrane it was thus co-purified with native acylated SLN^15^. To decipher the role of SLN on SERCA1a, we perform the same proteolytic experiment but using recS1a, in the absence or supplemented with chemically synthesized palmitoylated SLN (Fig. 3A,B, respectively). Considering that recS1a is purified from yeast cells in the presence of the detergent dodecylmaltoside (DDM), we first look for proteolysis conditions compatible with the presence of detergent. Therefore, a first series of experiments was done on the delipidated SR after solubilization by DDM. Proteolysis profiles of DDM-solubilized SR were similar to that obtained for native membrane embedded SERCA1a (Supplementary Fig. 1C) even though DDM significantly accelerates proteolysis as profiles obtained after a 5 min incubation were similar to that obtained after 60 min when the protein is embedded in its native lipids (Supplementary Fig. 1B). This acceleration is not the consequence of a loss of stability of the solubilized recS1a as depicted by PrtK-free samples incubated for 30 min (Fig. 3, right panels) but is rather due to an increased local dynamic of loops connecting the transmembrane domain to the A-domain, leading to a higher accessibility of the proteinase K cleavage sites. Note that Tg must be added to stabilize the detergent-solubilized ATPase as the sole addition of EGTA to remove calcium may result in a quick irreversible inactivation and aggregation of the ATPase^27^.

When palmitoylated SLN was added to recS1a (Fig. 3B), proteolysis was drastically slowed both in the presence of calcium (Fig. 3A,B, lanes 1–3) and in the presence of thapsigargin (Fig. 3A,B, lanes 4–6), indicating that SLN interacts with the Ca_2_.E1 state and also with the E2.Tg state. This observation is in agreement with the crystal structures of the complex obtained at 3 Å resolution^3,4^ and more recently with cross-linking experiments showing an interaction of SLN with SERCA1a at all the major steps of the catalytic cycle^11^.

### Sarcolipin slows E2 to E1 transition by moderately affecting binding of calcium

**Figure 4:**
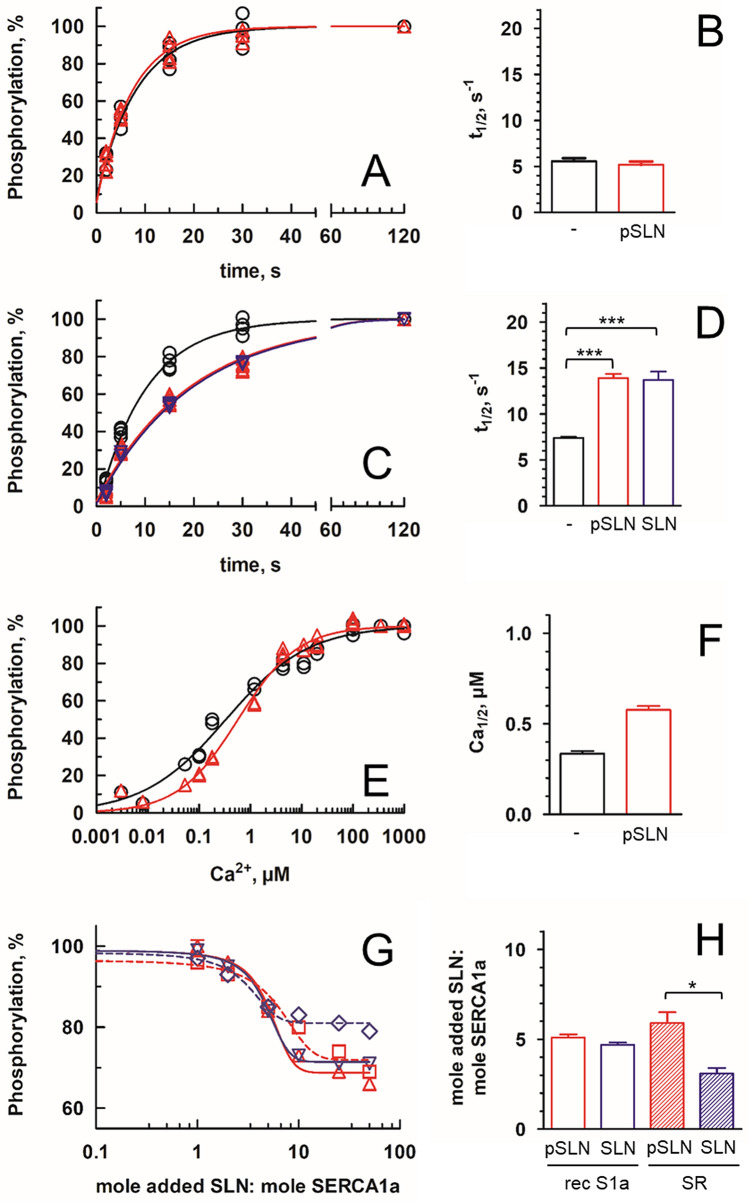
Rate of phosphorylation of recombinant SERCA1a in the presence of SLN. Kinetics of phosphorylation of the recombinant enzyme (recS1a), starting either from a calcium bound state (**A**) or a calcium deprived state (**C**). In each case, the maximum level of phosphorylation reached is indicated as 100%. recS1a was incubated in the absence (black circles) or in the presence of chemically synthesized pSLN (red triangles) or unacylated SLN (SLN, upside-down blue triangles) (**C**) at a Sarcolipin:SERCA1a of 10:1 (mol:mol). Rates of phosphorylation deduced from (**A**) and (**C**) are given as t_1/2_ on (**B**) and (**D**), respectively. Data were fitted following a one-phase association law. (**A**,**B**) Maximum level of phosphorylation (EPmax) corresponds to 3.37 ± 0.35 and 3.23 ± 0.37 nmoles EP/mg ATPase for recS1a and recS1a + pSLN with t_1/2_ = 5.7 and 5.1 s^−1^, respectively. Data are the average of four replicates from two independent experiments. p = 0.45 indicating that the two conditions are not significantly different. (**C**,**D**) EPmax corresponds to 3.09 ± 0.10, 3.10 ± 0.09 and 3.15 ± 0.05 nmoles EP/mg ATPase for recS1a, recS1a + pSLN and recS1a + SLN, respectively. The corresponding rate of phosphorylation are t_1/2_ = 6.8, 13.5 and 13.7 s^−1^, respectively. Data are the mean ± standard of six, six and four replicates from two independent experiments, respectively. p < 0.0001 for comparison of experiments done in presence of pSLN or SLN *vs* in their absence. (**E**,**F**) Phosphoenzyme level was assayed in the presence of varying amounts of calcium. Data were fitted with a one-site specific binding law with Hill slope. Data are the average of five independent experiments resulting in 36 data points per fit. EPmax corresponds to 3.13 ± 0.11 and 3.19 ± 0.11 nmoles EP/mg ATPase for recS1a and recS1a + pSLN with Ca_1/2_ = 0.33 ± 0.03 and 0.58 ± 0.05 µM with nH = 0.54 ± 0.02 and 0.74 ± 0.03, respectively. p = 0.29 indicating that the two conditions are not significantly different. (**G**,**H**) Effect of SLN:SERCA1a stoichiometry on the level of phosphorylated intermediate. (**G**) Phosphoenzyme level was assayed after 20 s phosphorylation in the presence of varying amounts of pSLN or SLN. recS1a + pSLN (red triangle), recS1a + SLN (upside down blue triangle), DDM-solubilized SR + pSLN (red square), DDM-solubilized SR + SLN (blue diamonds). The mean of three independent experiments is plotted. Error bars correspond to standard deviations and when small are hidden by symbols. EPmax are 2.37 ± 0.05, 2.38 ± 0.05, 4.00 ± 0.14 and 3.94 ± 0.12 nmoles EP/mg ATPase, respectively. Values obtained for recS1a and SR in absence of SLN were taken as 100%. The data were fitted to an inhibitory dose–response equation with variable slope. The amplitude of the inhibition is 31.2 ± 0.8, 28.6 ± 0.5, 28.1 ± 1.2 and 19.0 ± 0.7%, respectively. (**H**) SLN:SERCA1a ratio corresponding to an half-inhibition as deduced from (**C**). Half inhibition is achieved for 5.1 ± 0.3, 4.7 ± 0.2, 5.9 ± 0.6 and 3.1 ± 0.3 mol SLN added per mole of SERCA1a, respectively, with p = 0.047 for comparison of DDM-sol SR + pSLN and DDM-sol SR + SLN.

Since the pioneering work of MacLennan and coll. in 1998^23^, it has been admitted that SLN, as well as its cardiac homolog phospholamban (PLB), are moderately reducing apparent affinity for calcium (from 0.35 µM in absence of SLN to 0.51 µM in its presence). Effect on the turnover is less clear as it seems to highly depend on the system used for functional characterization. Whereas some authors observed a significant increase of the Vmax up to 140% of the Vmax measured in absence of SLN, others showed a clear decrease of the maximal rate of hydrolysis to about 70–80% in the presence of SLN (see^13^ and Supplementary Table 1). Structures of SERCA1a:SLN complexes^3,4^ showed an interaction of SLN with SERCA1a in an E1-like state probably preceding calcium binding. Therefore, we aimed at measuring intermediate reaction rates to dissect SERCA1a catalytic cycle. We focus on the E2 to Ca2.E1 transition by measuring rates of phosphorylation from [γ^32^P]ATP. As showed in Fig. 4A,B, the rate of phosphorylation of the recS1a in the absence or in the presence of pSLN is the same when starting from a calcium bound state (Ca_2_.E1), suggesting that pSLN does not affect the Ca_2_.E1 ~ P phosphoenzyme formation.

To specifically investigate the effect of SLN on the binding of calcium, we also evaluated the rate of phosphorylation when starting from a Ca^2+^-deprived state. We know that the detergent-solubilized ATPase can be stable for a few minutes in absence of calcium^27,34^. Samples were supplemented first with EGTA to reach nanomolar concentration of free calcium, followed by simultaneous addition of [γ^32^P]ATP and calcium to initiate phosphorylation. Interestingly, when starting from a calcium-deprived state (most probably a E1-like state at pH 7 as previously shown^35^) the rate of phosphorylation is significantly lower in the presence of SLN or pSLN (Fig. 4C,D). This effect indicates that the presence of SLN slows down the transition from the calcium-deprived state to the calcium-bound state. In addition, this inhibition does not depend on the acylation of SLN. Furthermore, the same level of phosphorylate intermediate (EP) is reached in absence and presence of SLN (3.09 ± 0.10, 3.10 ± 0.09 and 3.15 ± 0.05 nmoles EP/mg ATPase, in absence of SLN, or presence of pSLN or SLN, respectively) and the kinetics are monophasic suggesting that most of the ATPases interact with SLN.

Noteworthy, the rate of phosphorylation of a calcium-deprived DDM-solubilized native SR (Supplementary Fig. 5A,B) is slower than that of the recS1a enzyme alone and faster than that of the enzyme in the presence of SLN or pSLN. These particular series of experiments were done with a 1:10 mole:mole recS1a:SLN ratio. The recS1a:SLN ratio varied also from 1:1 to 1:50 (Fig. 4G,H). In that experiment, the amount of phosphorylated intermediate obtained 15 s after addition of [γ^32^P]ATP was measured. Maximum inhibition is in fact reached for a recS1a:SLN ratio of 1:10, strongly suggesting again that most of the ATPases interact with a SLN at such a ratio. Although this ratio is higher than the 1:1 to 1:2 ratio reported for native membrane^15,23^, it is in agreement with experiment available in the literature. As itemised in Supplementary table 1, ratios from 1:2 to 1:50 were investigated either in membrane or in detergent demonstrating that high ratios are necessary to clearly detect an interaction of SLN with SERCA1a that affects SERCA1a activity. Remarkably, higher SERCA1a:SLN ratios when using the recombinant enzyme or the native enzyme yield different results depending on the acylation state of SLN (Fig. 4G,H).

The rate of dephosphorylation in the absence or presence of pSLN was also measured. No effect of SLN, either palmitoylated or not, was detected after addition of non-radioactive ATP to trigger dephosphorylation (Supplementary Fig. 5C,D).

To measure the impact of pSLN on the binding of calcium, we first used intrinsic tryptophan fluorescence to estimate Ca_1/2_ at equilibrium as described previously for the detergent-solubilized recombinant SERCA1a^27^. A very small shift of the Ca_1/2_ was visible, with Ca_1/2_ varying from 2.9 µM in absence of pSLN to 4.5 µM in its presence (Supplementary Fig. 6A,B). In addition, we used an enzyme-coupled assay to estimate apparent affinity for calcium and turn-over rates. Again no difference was observed for the recS1a in the absence or in the presence of pSLN both on affinity for calcium and on maximum turn-over (Supplementary Fig. 6C,D). As SLN slows down the E2 to Ca2.E1 transition without affecting the turnover rate, it suggests that the dephosphorylation step remains the rate limiting step of the cycle even in the presence of the regulatory peptide, suggesting a role of SLN restricted to the calcium binding step. Therefore, to investigate the apparent affinity for calcium, we estimated the amount of phosphorylated intermediate after 15 s in the presence of varying concentrations of calcium (Fig. 4E,F). A small shift of the Ca_1/2_ from 0.33 to 0.58 µM was observed in the presence of SLN. Interestingly, we observed on both phosphorylation and calcium binding (Suppl. Fig. 6A) that pSLN slightly reduces affinity for calcium and to some extent increases cooperativity (nH). These effects are weak but in fair agreement with previously published data^8,13,23,36^. This increase of the cooperativity in the presence of SLN suggest that SLN does not affect the two calcium-binding sites in the same manner as proposed recently by molecular dynamics^37^.

### Sarcolipin impairs the binding of ATP and improves the binding of vanadate

**Figure 5:**
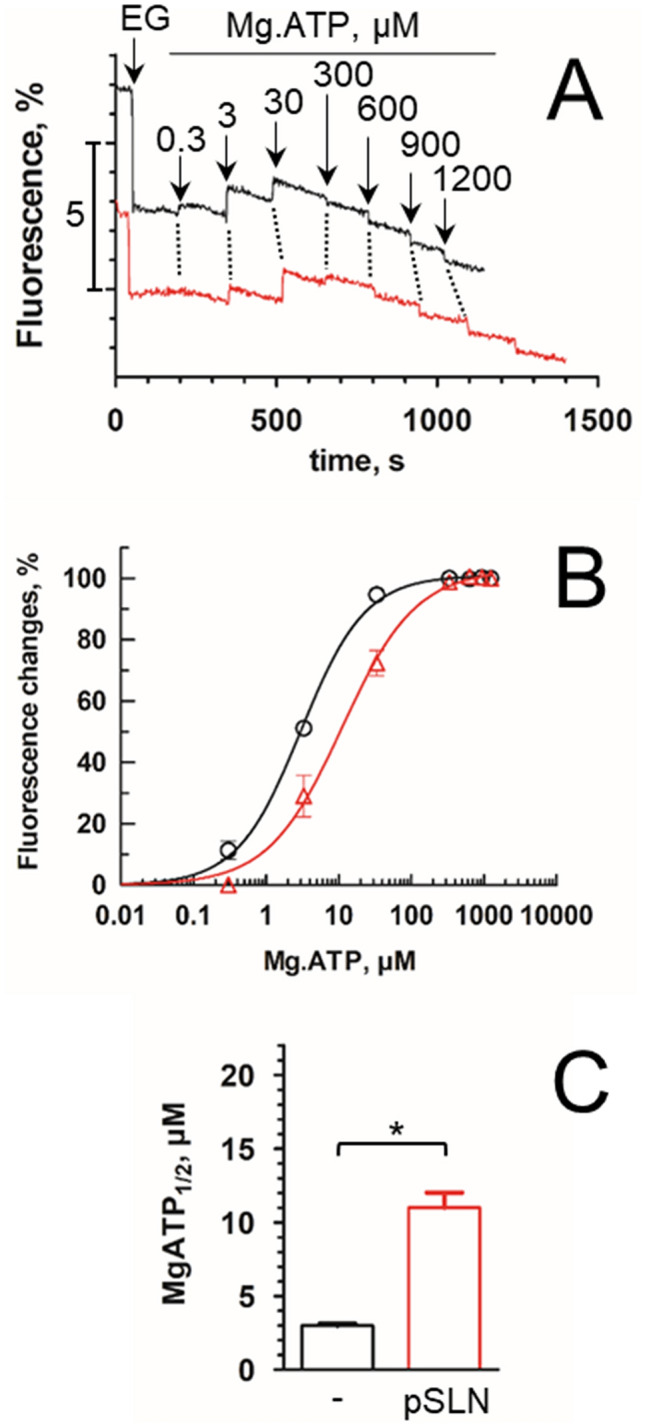
Effect of SLN on ATP binding. Binding of Mg.ATP as deduced from tryptophan fluorescence changes for recombinant SERCA1a alone (black) or supplemented with pSLN (red). (**A**) One representative series of experiments among three for each conditions are shown here. A first addition of 2 mM EGTA (EG) was done to reduce free Ca^2+^ to about 4 nM. Increasing amounts of Mg.ATP were added from 0.3 to 1200 µM. Larger additions of 300 µM Mg-ATP (from 600 to 1200 µM) led to a decrease of the fluorescence. Such additions were done to estimate the inner filter effect due to nucleotide absorbance. Note that using 295 nm instead of a shorter excitation wavelength minimizes the inner-filter effect of the nucleotide. Traces were corrected for small dilution effect upon each addition but not for inner filter effect of nucleotide. (**B**) The changes in fluorescence were plotted as fractional values after normalization to 100% of the maximal change in fluorescence in each series (recS1a, black circles; recS1a + pSLN, red triangles). Each traces are the average of three independent experiments. Data were fitted with a “one-site specific binding law” with Hill slope. nH are 0.99 ± 0.04 and 0.95 ± 0.06, respectively, thus in agreement with a unique binding site. (**C**) Affinity for Mg.ATP as deduced from (**B**). Mg-ATP_1/2_ are 3.00 ± 0.12 µM for recS1a and 11.0 ± 1 µM for recS1a + pSLN, with p = 0.045.

In the Albers-Post scheme, binding of calcium occurs after a conformational change during the transition from an E2 ground state, which cannot bind calcium, to an E1 state with a high affinity for calcium (Fig. 1)^1^. Considering the slowdown of the rate of phosphorylation from a calcium-deprived state (Fig. 4C,D), we wonder whether this inhibition is due to an inhibition of the transition from the E2 to the E1 state (E2 to E1), or to an inhibition of the binding of calcium (E1 to Ca_2_.E1). Binding of ATP and nucleotide analogues on calcium-deprived SERCA1a can be useful for probing the E2 to E1 equilibrium^38^. ATP is typically able to bind to the calcium-deprived E1 state and its presence accelerates binding of calcium^39,40^. Structures of the SERCA1a-SLN complex were obtained in the presence of AMPPCP^3^ or TNPAMP^4^, two non-hydrolysable ATP analogues. As these two molecules induce themselves subtle local reorganization of the nucleotide binding pocket, the authors could not conclude for any specific effect of SLN on the nucleotide binding site in presence of such analogues. To investigate whether SLN impacts the binding of nucleotide, we studied the effect of Mg.ATP on the tryptophan fluorescence emission of the DDM-solubilized and glycerol-stabilized SERCA1a in the absence or presence of pSLN (Fig. 5). Effect of pSLN was clearly observed on raw data (Fig. 5A). While an addition of 0.3 µM Mg.ATP induces an increase of the fluorescence level in absence of pSLN, no fluorescence intensity variation is observed in the presence of pSLN. Additionally, a concentration of 300 µM of Mg.ATP induces a decrease of the fluorescence level in absence of pSLN whereas such a concentration of Mg.ATP induces an increase of signal in presence of pSLN. It indicates that a concentration of about 30 µM Mg.ATP is sufficient to saturate the SERCA1a site in absence of SLN but not in its presence. Affinity of the recS1a for Mg.ATP was 3 µM, but significantly drops to 11 µM in the presence of pSLN (Fig. 5B,C). Such an increase of the K_Mg.ATP_ indicates that SLN affects the binding of nucleotide and shifts the E2 to E1 equilibrium towards E2.

**Figure 6:**
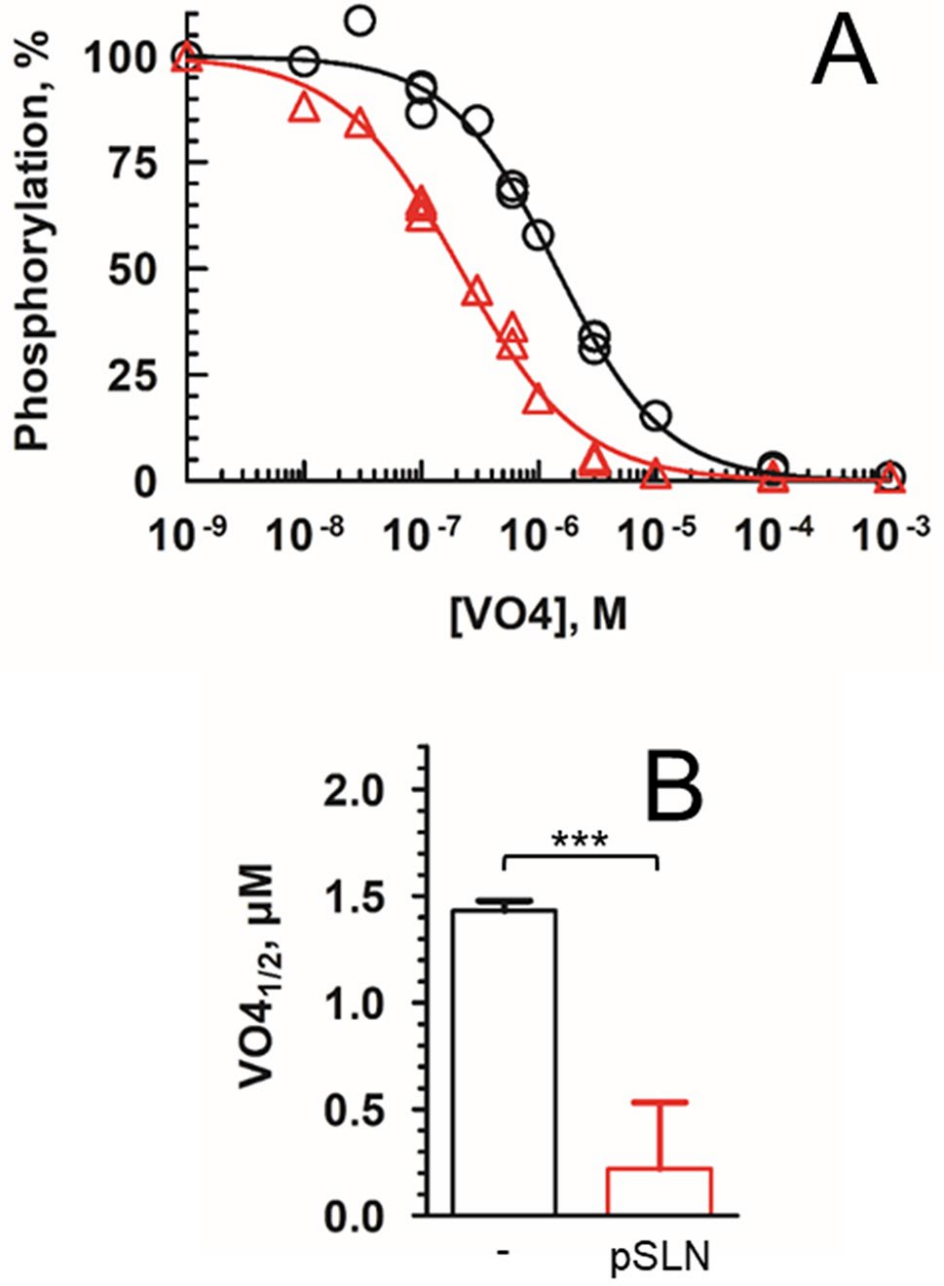
Effect of SLN on the binding of orthovanadate. The amount of phosphorylated intermediate was assayed after preincubation with various concentrations of orthovanadate, for the recombinant enzyme alone (black circles) or supplemented with pSLN (red triangles). (**A**) The maximum level of phosphorylation reached in each case is indicated as 100%. EPmax were 2.01 ± 0.05 and 2.07 ± 0.21 nmoles/mg of ATPase, respectively. All the data points are shown and are from three independent experiments. Data were fitted with a “one-site specific binding law” with Hill slope. nH are 0.99 ± 0.04 and 0.95 ± 0.06, respectively, thus in agreement a unique binding site. (**B**) Apparent affinity for vanadate as deduced from panel A. K_1/2_ are 1.43 ± 0.08 µM and 0.22 ± 0.01 µM, respectively, with p < 0.0001.

Vanadate is a well-known inhibitor of P-type ATPase which preferentially binds to the E2 state (Fig. 1). It mimics an intermediate state of the dephosphorylation by occupying a site resembling to that of phosphate near the catalytic aspartate^41^. Therefore, an increase of the apparent affinity for vanadate indicates that the E2 to E1 equilibrium is pulled toward E2 while a decrease of this apparent affinity indicates a shift of the equilibrium toward E1. Thus, to evaluate whether SLN shifts the E2 to E1 equilibrium, we also measured the binding affinity of vanadate to recS1a in presence or absence of pSLN. The enzyme was first equilibrated in presence of varying concentration of vanadate at 20 °C. Then, the amount of vanadate-free enzyme was determined by addition of calcium and [γ^32^P]ATP simultaneously to trigger phosphorylation. Consequently, the amount of phosphoenzyme measured corresponds to the amount of vanadate-free enzyme available before addition of calcium and [γ^32^P]ATP. The K_0.5_ value for vanadate inhibition is 1.43 ± 0.08 µM for DDM-solubilized recS1a in absence of pSLN (Fig. 6). This value drops significantly to 0.22 ± 0.01 µM in the presence of SLN suggesting an increased apparent affinity for vanadate of SERCA1a when SLN is bound. Thus, it confirms that SLN is not only impairing calcium binding as mentioned above (Fig. 4) but also hampers the transition toward the calcium high affinity state E1, as suggested by the concomitant lowering of the affinity for Mg-ATP (Fig. 5). Inhibition by vanadate was also measured for DDM-solubilized SR by the same method (Supplementary Fig. 7). The K_0.5_ value for vanadate inhibition is 0.54 ± 0.03 µM for DDM-solubilized SR, a value comprises between the value obtained for recS1a alone and that obtained for recS1a in presence of SLN, as observed for rate of phosphorylation previously (Supplementary Fig. 5A,B).

## Discussion

Our data show that chemically synthesized SLN interacts with recombinant SERCA1a, with calcium-deprived E2 state as well as with calcium-bound E1 state. SLN has an allosteric effect on SERCA1a transport activity by impairing the binding of ATP. Our results reveal that SLN significantly slows down the E2 to Ca_2_.E1 transition of SERCA1a while it affects neither phosphorylation nor dephosphorylation. Comparison with chemically synthesized SLN deprived of acylation demonstrates that palmitoylation is not necessary for either inhibition or association with SERCA1a but can affect SERCA1a phosphorylation.

The role of Sarcolipin on the regulation of SERCA1a at molecular level is a long-standing question. Careful analysis of published data on biochemical characterization of regulatory peptides, especially SLN, highlights a heterogeneity of the results^13^, perhaps because most of the studies were done without taking into account the animal species, the presence of some endogenous regulatory peptide when starting from native sources, or the presence of post-translational modifications which varies among species^15^. As mentioned in introduction, even though these studies may have not been compromised by these uncontrolled parameters, they need obviously to be re-evaluated now taking into account recent inputs especially on the effect of mild detergent on the interaction between SLN and SERCA1a. While most of the results were obtained after reconstitution of native SERCA1a solubilized with mild detergent such as C_12_E_8_ or DDM, we demonstrated a few years ago by combining Size Exclusion Chromatography and MALDI-TOF analysis that mild detergents are unable to disrupt native SERCA1a:SLN complexes^15^. As a consequence, all previous studies following such an approach should have taken into account the presence of some native SLN, about 10 to 30%, depending on the method of purification (Supplementary Table 1 and references herein). Here, we designed an experimental procedure aiming at studying the effect of SLN in conditions where these parameters are controlled: we studied the role of rabbit SLN on rabbit SERCA1a. Rabbit SERCA1a was expressed in *S. cerevisiae*, i.e. in the absence of endogenous SLN, following the procedure we developed a few years ago^30^. Rabbit palmitoylated and unacylated SLN were chemically synthesized in accordance with our recent procedure^29^. As reconstitution protocols do not always allow an accurate control of protein orientation, we choose to work with solubilized forms as detailed in the Supplementary information, assuming that mixing of solubilized SLN (palmitoylated or not) with solubilized SERCA1a will allow the two proteins to interact properly as previously shown by NMR in the presence of dodecylphosphocholine^26^. The use of a 1:10 SERCA1a:SLN mole:mole ratio was enough to get maximum effect of chemically synthesized SLN on the ATPase (Fig. 4G,H). Furthermore, the effective SLN:SERCA1a is likely to be low and most probably close to 1 as deduced from calculations (see “Methods” section), a ratio in fair agreement with other experiments described in the literature (Supplementary table 1), and probably close to the actual molar ratio in SR membranes^23,42^. Interestingly, such heterogeneity in the results was also mentioned for PLB by H. Young and coll. as discussed previously^25^ indicating that careful attention have to be taken for the design of protocols.

The Nissen and Toyoshima groups reported simultaneously in 2013 the first two and only crystal structures of a SERCA1a/SLN complex^3,4^. These complexes were obtained in the presence of 75 mM and 40 mM magnesium, respectively, a concentration significantly higher than the one expected in vivo (from 0.5 to 4 mM depending on the method used^43^). Such high concentrations are enough to fully saturate magnesium binding sites on the ATPase, either the catalytic site in the cytosolic domain or the calcium binding sites in the transmembrane domain^44^. Based on these crystal structures, the authors hypothesised that SLN stabilizes an E1.Mg state. However, effect of magnesium on the E2 to Ca_2_.E1 equilibrium has been extensively studied for 40 years, demonstrating that magnesium itself is enough to stabilize an E1.Mg state, whether SERCA1a is from native SR or from recombinant source^27,35^. Additionally, recent in silico studies have shown that the E1.Mg state is likely a non-physiological state that is not favored in the cell, and that SLN, as phospholamban, may populate a metal ion-free intermediate state^45^. In summary, these results strongly suggest that the E1.Mg state as illustrated by the published SERCA1a/SLN structures is indeed not a physiological intermediate in the catalytic cycle and probably not a SLN-inhibited state, even if SLN remains associated with this conformation despite the presence of metals as magnesium in the published structures^11^.

Recent molecular dynamics studies have also provided insight in the impact of SLN on SERCA1a, notably on the calcium binding sites. The presence of SLN perturbs the Ca^2+^ binding sites occlusion at E309, leading to an increase in the distance E771-D800, which produces incompetent Ca^2+^ binding sites and allows Ca^2+^ backflux to the cytosol^37^. These results are in agreement with the increase of cooperativity we observed (Fig. 4E and Supplementary Fig. 6A). A tougher binding of calcium at site one, i.e. between D800 and E771, may indeed result in an increase of the cooperativity^46,47^. In addition to subtle perturbations of the calcium binding sites architecture, we observed a poorer affinity for Mg.ATP in presence of SLN (Fig. 5). Such long-distance effect of SLN was never mentioned before. It demonstrates that SLN acts as an allosteric regulator both on calcium and ATP binding sites, thus subtly perturbing the interdomain communication that usually ensure efficient calcium transport. This long-distance effect of SLN was recently detailed at molecular level using molecular simulations. In particular, a subtle straightening of M5 occurs in presence of SLN which leads to a reorientation of the loop containing the DKTG catalytic motif, thus perturbing binding of Mg.ATP^48^ (*in press*).

A few years ago, we demonstrated using mass spectrometry that rabbit SLN is S-acylated on its only cysteine (Cys9) with a palmitate or an oleate. No electronic density could be assigned to a palmitate or oleate chain on the electronic density map of the crystal of SERCA1a-SLN complex^3,4^ whereas this chain is undoubtedly linked to the peptide, the samples being prepared rigorously in the same conditions^15^. This post-translational modification is also present in pig SLN and presumably in a few other mammals and birds, as judged from the amino-acid sequences^15^. Among the other hundred sequences aligned covering most of the animal kingdom, other species including human present a phenylalanine instead of a cysteine at position 9 (Supplementary Fig. 2A,B) suggesting that acylation is missing for these organisms, and thus acylation is not critical for enzyme regulation in these organisms. Here, we confirmed that palmitoylation is indeed not necessary for either association with or inhibition of SERCA1a (Figs. 3 and 4, respectively), especially during the transition from the E2 ground state to the Ca_2_.E1 state. A few years ago, we also showed that deacylation of native SLN, obtained after treatment with hydroxylamine, had no effect both on the affinity for calcium at steady state and on Vmax, either in membrane or after solubilization by C_12_E_8_^13^.

We observed that a DDM-solubilized SR sample has an intermediate rate of phosphorylation compared to the purified recS1a in the absence and in the presence of chemically synthesized SLN (Supplementary Figs. 5A,B and 7). As we demonstrated in 2014, most of the native ATPase remains associated to SLN even after solubilisation in the presence of large amount of mild detergent and purification by size exclusion chromatography, conditions necessary to provide monomeric SERCA1a^15^. However, the persistence of native lipids after such a purification could account for such an intermediate rate of phosphorylation or inhibition by vanadate compared to the recombinant enzyme that is certainly deprived of any lipids after purification. Detergent to lipid ratios can affect the equilibrium at several steps of the catalytic cycle, especially during the transition from the E2 to the Ca_2_.E1 state^49^. Noteworthy, we demonstrated that addition of chemically synthesized SLN on DDM-solubilized SR led to an increased inhibition of the rate of phosphorylation (Fig. 4G,H), showing that most but not all, of the ATPase are associated with native SLN despite the fact that it already contains 1:1 to 2:1 pSLN/SERCA1a mole/mole^15,23^. We also demonstrated that palmitoylation itself has an effect on the degree of the inhibition when tested on SR (Fig. 4G,H). By contrast, inhibition of recS1a, was the same by Palm-SLN or SLN (IC50 ~ 5.0 ± 0.2 and 4.5 ± 0.2 mol of added SLN/mole of SERCA1a, respectively, and a maximum decrease of about 30% of the EP level). Surprisingly, native SERCA1a is not inhibited to the same extent according to the palmitoylation of SLN. In the presence of pSLN, inhibition of DDM-solubilized native SERCA1a is similar to that of recS1a (IC50 ~ 5.6 ± 0.7 mol added SLN/mole of SERCA1a with a decrease of about 30% of the EP level). In the presence of unacylated SLN, inhibition is now weaker with only a decrease of about 20% of the EP level. Unexpectedly, the IC50 is only about 2.9 ± 0.3 mol added SLN/mole of SERCA1a indicating that less SLN are necessary to inhibit the ATPase when it is not palmitoylated, suggesting that binding of unacylated SLN to native SERCA1a is more efficient.

Besides its various roles in protein signalling and addressing, palmitoylation can also regulate oligomerisation processes of membrane proteins^50^. Recent work has shown that other P-type ATPases regulatory subunits like phospholamban (PLB) and phospholemman (PLM) are palmitoylated^16,17^. Acylation of PLB and PLM is functionally relevant by having an impact on the oligomerisation of the peptides, their association with their dedicated pumps (Ca^2+^-ATPase or Na+, K+-ATPase, respectively) and on their lifetime^18^. PLB pentamerization is now clearly demonstrated by molecular dynamics simulations, and by solid state or solution NMR^51–53^. PLB pentamers act as reservoir and dissociation into monomers leads to the inhibitory function^54^. Interestingly, palmitoylation of PLB:Cys 36 (Supplementary Fig. 2A,C) increases affinity for protein kinase A (PKA) and reduces affinity for protein phosphatase 1a (PP1a), resulting in an increased level of phosphorylation of PLB. In addition, oligomeric PLB is mainly palmitoylated while monomeric PLB is much less so^16^. PLB phosphorylation results in a release of SERCA1a inhibition without its full dissociation from the pump^14^. All these data on PLB could suggest that the same kind of interplay between SLN oligomerisation, palmitoylation and phosphorylation for inhibition of SERCA1a is present. Oligomerisation of SLN was recently proposed as a mechanism for SERCA1a regulation relying on the reduction of the interaction of both proteins similarly to PLB^55,56^. Even if the data presented in Fig. 4G,H require further investigation, they are statistically significant and they suggest that palmitoylation of SLN could affect SLN oligomerisation. PLB:Cys 36 is a residue predicted to be at the membrane interface as SLN:Cys9^3, 15^. Sarcolipin also forms oligomers in detergent micelles, liposomes^57^ and membranes^56^. Phosphorylation of SLN:Thr5 results in a loss of inhibition of SERCA1a^58^. Although the functional link between oligomerisation, palmitoylation and phosphorylation of SLN has not been demonstrated, considering the analogy with PLB, a similar mechanism of regulation of SLN could exist. Indeed, we demonstrated a few years ago from sequence analysis, in particular DNA sequences, that a few mammals and birds share mutation of Phe 9 to Cys 9^15^. This mutation does not correspond to a same codon. Mammals have indeed a different codon for Cys than birds. In addition, we observed that the mutation from Phe to Cys was not present in the last common ancestor of these two groups as deduced from cladogram analysis. All these results are in agreement with an independent and recurrent evolution designated as convergent evolution. As a PTM is energetically costly for cells, the fact that a few organisms conserved or acquired it, strongly suggests that this modification has an important role, even if this role is restricted to some species and not present in human. Incidentally, PLB:Cys 36 is also only present in 70% of the species indicating that PLB palmitoylation does not exist in all the organisms, being replaced by a Thr (25%), a Ser (~ 3%) or a Phe (~ 1%) residue (Supplementary Fig. 2C). Although the experimental data presented here are simply not as strong as those data that support the mechanistic significance of PLM or PLB palmitoylation, more experiments in the future might enlighten the role of the palmitoylation of SLN.

In summary we demonstrate for the first time the impact of SLN on partial reaction of the enzymatic cycle of SERCA1a: SLN slows down the E2 ground state to E1 state transition impairing the binding of calcium; the rate of phosphorylation (Ca2.E1 to Ca_2_.E1 ~ P) and the rate of dephosphorylation (Ca_2_.E1 ~ P to E2) of SERCA1a are not affected by SLN. Surprisingly, we observe an impact of SLN on nucleotide and vanadate binding suggesting that SLN not only prevent calcium binding as early proposed but also disrupts interdomain communication. While palmitoylation appears not to be essential for calcium binding and turn-over, it has a small but statistically significant effect on SERCA1a phosphorylation when various ratios of SLN:SERCA1a or PalmSLN:SERCA1a are tested. If the role of SLN is now better understood, more studies are needed to decipher the role of post-translational modifications of SLN in relation to SERCA1a activities.

## Methods

### SR membranes and chemicals

Sarco-endoplasmic Reticulum (SR) membranes were prepared from rabbit muscles as previously described^27^. Therefore, all experiments and methods were performed in accordance with relevant guidelines and regulations, in strict accordance with the recommendations and after agreement from the Ethic committee of the “Commissariat à l’Energie Atomique et aux Energies Alternatives” (CEA agreement #E 91 272 106). Octaethylene glycol mono-n-dodecyl ether (C_12_E_8_) was purchased from Nikkol Chemical (#BL-8SY; Tokyo, Japan), and n-dodecyl β-d-maltopyranoside (DDM), from Anatrace (#D310; Maumee, OH). Streptavidin Sepharose high performance resin was provided by GE Healthcare (#17-5113-01; Orsay, France). Thapsigargin (TG stock solution was 1 mg/mL in DMSO, i.e., about 1.5 mM) was from VWR International (#586005; Fontenay-sous-Bois, France). [γ^32^P]ATP was from Perkin-Elmer (#BLU002A-250UC; Courtaboeuf, France). All other chemical products were purchased from Sigma (Saint-Quentin Fallavier, France). Sequences for SERCA1a heterologous expression and for SLN chemical synthesis were both from rabbit.

### Proteinase K treatment

Yeast-expressed and purified SERCA1a (recS1a) was supplemented with SLN at a mole:mole ratio of 1:10 recS1a:SLN. In absence of SLN, recS1a was supplemented with 0.07 mg/mL C_12_E_8_, an amount that corresponds to the detergent brought with chemically synthesized SLN. After 30 min at 20 °C, samples were supplemented with either 1.1 mM EGTA (Ca^2+^_free_ ~ 1 mM), or 50 mM EGTA (Ca^2+^_free_ ~ 150 nM) and 0.01 mg/mL Tg. After an additional incubation for 10 min at 20 °C, proteolysis was achieved by addition of 0.3 mg/mL proteinase K. Proteolysis was stopped by addition of 1 mM PMSF and incubation on ice for 10 min. After proteolysis arrest, samples were diluted twice in a 4 M-urea containing denaturation buffer, boiled for 60 s, and about 0.75 µg protein were loaded on a 9% SDS-PAGE prepared in the presence of 1 mM Ca^2+^^32^. After electrophoresis, gels were stained with Coomassie blue.

### Tryptophan fluorescence measurements

Intrinsic fluorescence of SERCA1a was measured with a SPEX Fluorolog spectrofluorometer (Horiba/Jobin–Yvon, Longjumeau, France) as described before^27^. Briefly, signals were monitored with excitation and emission wavelength set at 295 and 320 nm, with bandwidths of 2 and 10 nm, respectively. Changes in tryptophan fluorescence were recorded at 20 °C after dilution of protein samples to a final concentration of about 6–7.5 µg/mL into a medium containing 50 mM MOPS-Tris pH 7, 100 mM KCl, 5 mM MgCl_2_, 20% glycerol (v/v) and 10 mg/mL DDM. In presence of SLN, recS1a and SLN were first mixed together and incubate for 1 h on ice before measurement. In absence of SLN, recS1a is supplemented with 0.07 mg/mL C_12_E_8_ to compensate the detergent present with SLN. The Maxchelator program was used for estimating [Ca^2+^]_free_^59^ (program available at https://somapp.ucdmc.ucdavis.edu/pharmacology/bers/maxchelator/downloads.htm). The dissociation constant of the Ca.EGTA complex was taken to be 0.4 µM at pH7 and was assumed to be the same in the presence of glycerol.

### Phosphoenzyme formation from [γ^32^P]ATP and turnover-dependent dephosphorylation

Transient formation of the phosphoenzyme intermediate was measured after incubation with [γ^32^P]ATP followed by acid quenching, and filtration. For most experiments, 50 µL of enzyme at 10 µg/mL in a buffer containing 50 mM Tes-Tris pH7.5, 1 mM KCl, 5 mM MgCl_2_, 20% glycerol (w/w) and 0.5 mg/mL DDM, supplemented or not with SLN, was stored on ice. Concentrations of EGTA and calcium are specified in figure legends in order to reach the appropriate [Ca^2+^]_free_ and therefore the desired starting state for the ATPase. For kinetics of phosphorylation, we initiate reaction by addition of 2 µM [γ^32^P]ATP (5 mCi/µmole), and calcium at 100 µM when specified. Reaction was quenched by addition of 2 mL of cold quenching medium (500 mM TCA + 30 mM H_3_P0_4_) under vigorous mixing, and immediately stored on ice for 20–30 min to improve precipitation, a period of time critical for retention of the precipitate on the filter in presence of detergent^60^. This was followed by filtration on GS filters (Millipore, Saint Quentin-Fallavier, France) and careful rinsing of the filters with diluted quenching medium (50 mM TCA + 3 mM H_3_P0_4_). The amount of radioactivity bound to the filter was estimated by liquid scintillation counting (Perkin Elmer TriCarb Counter). When starting from a calcium-deprived state (Fig. 4C,D), EGTA was added only one minute before starting phosphorylation to limit irreversible inactivation of the solubilized ATPase, although the presence of glycerol helps in protecting the enzyme from irreversible inactivation^27^. For dephosphorylation kinetics, 1 mM “cold” Mg.ATP was added after a 30 s period of phosphorylation. For Ca^2+^-dependency, we adjust the concentration of free calcium according to the Ca.EGTA constant as mentioned above, before triggering phosphorylation. To determine vanadate binding affinity, the ATPase, supplemented or not with SLN, was incubated with various concentrations of orthovanadate on ice for 15 min in absence of calcium, before getting phosphorylated for 30 s. Phosphorylations were done at 0 °C especially for inhibition by vanadate to prevent dissociation of vanadate and limit irreversible inactivation in absence of calcium.

### Calculation of micelle/protein ratios

In most of our experiments, the concentration of DDM is 0.5 mg/mL i.e. about 1 mM, including about 0.15 mM accounting for monomers of DDM (Critical Micellar Concentration (CMC)) and about 0.85 mM assembled in micelles. As the aggregation number of DDM is about 80–120 molecules, the concentration of DDM micelle is about 7–11 µM in the samples. A SERCA1a monomer binds about 100–120 mol of DDM, so an amount of detergent corresponding more or less to a micelle^61,62^. SERCA1a is at 0.09 µM in most of the final samples. Therefore, when the chosen nominal ratio of SLN to SERCA1a is 10:1, the final ratio including detergent is about 80–120 micelles:10 SLN:1 SERCA1a (mole:mole:mole), indicating that the number of detergent-SLN-SERCA1a complexes is likely to be less than could be inferred at first view since a number of SLN could be present in the micelle–SLN complexes devoid of SERCA1a. Thus if we assume that the excess SLN gets distributed in empty micelles, for a given micelle, the SERCA1a:SLN ratio is likely to be much lower, maybe close to 1, a ratio close to the actual molar ratio in SR membranes as determined by MacLennan and coll. or others^23,42^. Nevertheless limited proteolysis assays demonstrate that interaction between SERCA1a and SLN occurs in such conditions (Fig. 3 and Supplementary Fig. A).

### Calculations and statistical analysis

Experiments were conducted at least thrice. The purified protein came from two independent batches of yeast membranes. Two batches of chemically synthetized pSLN and SLN were also compared giving strictly identical results. We analysed the complete set of data by nonlinear regression as mentioned in figure legends, and whenever possible, we submit data to statistical analysis with GraphPad Prism software. Average values are accompanied with standard deviation. Student one-tailed paired test was applied to calculate p-values. We consider a P > 0.05 as not significant.

## Supplementary Information

Supplementary Information.

## Data Availability

All relevant material is contained within the main text or supplementary information file.

## Abbreviations

C_12_E_8_: Octaethylene-glycol-dodecylether
DDM: β-Dodecyl maltoside
PLB: Phospholamban
SLN: Sarcolipin
PLM: Phospholemman
SR: Sarco-endoplasmic reticulum
SERCA1a: Sarco-endoplasmic reticulum Ca^2^^+^-ATPase, isoform 1a
recS1a: Recombinant yeast expressed SERCA1a
EGTA: Ethylene glycol-bis(2-aminoethylether)-N,N,N′,N′-tetraacetic acid
Tg: Thapsigargin
pSLN: palmitoylated Sarcolipin
