# Supplementary material for: Sarcolipin alters SERCA1a interdomain communication by impairing binding of both calcium and ATP
Source: Sci Rep. 2021 Jan 15;11:1641. doi: 10.1038/s41598-021-81061-6 (PMC7810697; doi:10.1038/s41598-021-81061-6)
Supplement: Supplementary file 1 — Supplementary Information. [file 41598_2021_81061_MOESM1_ESM.pdf]

Supplementary Informations for

Sarcolipin alters SERCA1a interdomain communication by impairing binding of both calcium and ATP

by Cédric Montigny, Dong-Liang Huang, Veronica Beswick, Thomas Barbot, Christine Jaxel, Marc le Maire, Ji-Shen Zheng, and Nadège Jamin

Supplementary informations

Considering the data provided by the literature (see Supplementary Table 1), one of our main concern was to control as much as possible both protein orientation to get properly assembled SLN:SERCA1a complexes, and SLN to SERCA1a ratio to favour specific interaction. It has been shown that, for membrane proteins not emerging from the membrane, i.e. deprived of large cytosolic domain as is the case for SLN, it can be particularly difficult to control the orientation after co-reconstitution ([1] and references hereafter). The final orientation of membrane protein in proteoliposomes was shown to really depend on the lipid nature, the detergent, the ionic strength, the pH, the membrane curvature, and the shape of the membrane protein [2–5]. Although the reconstitution of SERCA1a alone or in the presence of PLB leads to about 80±10% of right side oriented proteins as described by Rigaud and coll. and Young and coll. [6,7], this favoured orientation is most probably due to the large cytosolic headpieces of these proteins. Indeed, these large hydrophilic domains may not enter in the destabilized liposomes during reconstitution because of steric restrictions and/or electrostatic interactions with phospholipids [6], and for PLB, well-known interactions with the SERCA1a N-domain can undoubtedly help in orienting PLB towards the cytosolic leaflet [8–10]. SLN is clearly different from PLB as its N-terminus is very short (Supplementary Figure 2) and involved in a few interactions with SERCA1a compared to PLB [11–16]. Furthermore, in our hands, reconstitution of SERCA following the method of Rigaud and coll. leads to a 50/50 distribution of the SERCA orientation [17], indicating that fine-tuning of reconstitution parameters is certainly necessary. Taking all these points into account, co-reconstitution of recS1a in the presence of SLN would lead to an unknown amount of complexes properly formed and right-side-out oriented, therefore requiring additional cumbersome fine-tuning as well as additional assay to assess complexes formation and orientation.

We and others have shown over the last forty years that DDM- or $C_{12}E_8$-solubilized native or recombinant S1a are stable for long period from a few hours to days depending on the experimental conditions and that this solubilized enzyme behaves as the native SERCA1a [18–26]. Note that in the very last reference, yeast-expressed DDM-solubilized purified enzyme behaves as the recombinant SERCA1a in microsomes prepared from cos-7 cells. It indicates again that the lipid environment is not mandatory for measuring the turn-over rate and following the binding of ligands whether by intrinsic fluorescence or by measuring phosphorylation rates. Thus, we chose to use yeast-expressed and purified SERCA1a (recS1a) and synthesized SLN in the presence of DDM or $C_{12}E_8$. This strategy was already successful for structural analysis by NMR of the SLN:SERCA1a complex in dodecylphosphocholine [27]. We indeed assume that the dynamics of the protein-detergent complex enable the two partners to interact in the right orientation. The agreement between our experiment and others from the literature confirms that the interaction between SLN and SERCA1a occurs in spite of the presence of the detergent. Our protocol should be suitable for studying the role of other regulatory peptides on SERCA1a.

Supplementary methods

Use of synthesized Palm-SLN and SLN

The RBM^{GABA} strategy was used to synthesize peptide SLN(1-12,$E^{2,RBM}$,$C^{9,Palm}$)-SAL (**1**). In brief, the peptide is prepared on Rink amide ChemMatrix resin (0.1 mmol, degree of substitution: 0.45 mmol/g) by using the microwave-assisted fluorenylmethyloxycarbonyl (Fmoc)-based solid-phase peptide synthesis (SPPS) method. The general synthetic procedures include reductive amination, reduction of the nitro group on the RBM, introduction of the Lys_6 tag, protection of the 2-OH by GABA group on the RBM to tolerate the trifluoroacetic acid (TFA) cleavage, removal of the Mmt group on the Cys and palmitoylation. Please see [28] for details on materials provenance and procedure. After the assembly of the full-length peptide, the resin was treated with a TFA cocktail at room temperature to obtain 21 mg of crude peptide (**1')** (isolated yield: 11.1%). Finally, the GABA protecting group was removed under pyridine/AcOH (7:6, mol/mol) conditions to give the target peptide (**1)** (16.1 mg, isolated yield: 76.7%).

Peptide SLN (13-31, $L^{16, RBM}$) (**2)** was also synthesized using the RBM^{GABA} strategy. Wang ChemMatrix resin (0.1 mmol, substitution: 0.38 mmol/g) was used to synthesize using the microwave-assisted Fmoc SPPS method. The main steps in the synthesis include reductive amination, reduction of the nitro group on the RBM, introduction of the Lys_6 tag, protection of the 2-OH on the RBM by the GABA group. After that, the peptide was cleaved from the resin using TFA cocktails (TFA/H_2O/TIPS/ Phenol, 88/5/2/5, v/v v/wt) for 2 h to obtain 230 mg of crude peptide **2'**. The crude **2'** was then converted into crude peptide **2** at pH 6.9 (6 M Gn•HCl, 100 mM NaH_2PO_4) within 5 minutes. After RP-HPLC purification, the purified SLN (13-31, $L^{16, RBM}$) **2** (47.3 mg) was obtained as a white powder with an isolated yield of 20.6%.

N-terminal peptide **1** (11.2 mg, 4 μmol, 1 equiv.) and C-terminal peptide **2** (13 mg, 4 μmol, 1 equiv.) were dissolved in pyridine/acetic acid (1/6, mol/mol, 0.2 mL, 20 mM) to perform STL reaction for 4 h at 30ºC. The ligated product was purified by RP-HPLC to afford SLN (1-31, $E^{2, RBM}$, $L^{16,RBM}$, $C^{9, Palm}$) **3** (8.5 mg, 35% isolated yield). Then RBM-labeled peptide **3** (1 mg/mL, 3 mg) can be cleaved with 0.1 M HCl in HFIP and scavenger (1% TIPS) to obtain **S-Palm SLN** (1.6 mg, 53%). Peptide **3** (5 mg) was treated with 5% hydrazine hydrate for 30 min to remove S-palmitoylation and then freeze-dry the reaction solution. Then use 0.1 M HCl in HFIP and scavenger (1% TIPS) to remove RBM to obtain **SLN** (2.1 mg, 42%).

Reverse-phase chromatography profiles and ESI-MS analysis are available in Supplementary Figure 3 for peptides 1-3, respectively. Final products were lyophilized for long-term storage until being used.

Extreme care has to be taken for resuspension of hydrophobic peptides in detergent solution to prevent aggregation of peptides by hydrophobic, non-specific interactions in an aqueous environment. For Palm-SLN and SLN, we followed the procedure described in [29]. Briefly, SLN powder was initially

resuspended in a large volume of pure water and lyophilized to evaporate most of the TFA. The rinsed powder was then suspended in presence of 10 mM HCl to exchange the anionic residual TFA that may interact with the positive charges of the peptide for chloride. This suspension was lyophilized again. Three additional cycles of – dissolving in pure water / lyophilisation – will allow to eliminate traces of TFA and to get back to a pH compatible with functional tests. For final solubilisation of SLN in detergent, powder was then dissolved in 50% TFE and an amount of detergent to reach a 100:1 mole:mole detergent:SLN ratio. Here, we used $C_{12}E_8$ as it is fully compatible with functional characterization of SERCA1a [23]. This suspension was then lyophilized again to evaporate TFE. Sarcolipins (Palm-SLN or SLN) were finally resuspended at 0.8 mg/mL (about 200 µM) in a 10 mM potassium phosphate buffer pH 7.4, in the presence of 2.5 mg/mL $C_{12}E_8$.

Expression and Purification of recombinant SERCA1a (recS1a)

The yeast expression plasmid pYeDP60-SERCA1a-BAD (WT) and expression in *Saccharomyces cerevisiae* was described previously [30]. Yeast membranes were solubilized by DDM for subsequent purification of recombinant Ca^{2+}-ATPase by Streptavidin affinity chromatography [31]. Purified recombinant SERCA1a was recovered in a buffer containing 50 mM MOPS-Tris pH7, 100 mM KCl, 5 mM $MgCl_2$, 2.1 mM $CaCl_2$, 40 % glycerol (v/v) and 0.5 mg/mL DDM, together with some thrombin remaining from the elution procedure. The protein concentration in the purified fraction was in the 0.05 to 0.15 mg/mL range depending on the batch.

DDM-solubilized native SERCA1a purification

SR vesicles were suspended at a concentration of 1 mg/mL in a buffer containing 50 mM MOPS-Tris pH7, 100 mM KCl, 5 mM $MgCl_2$, 0.1 mM $CaCl_2$, 20 % glycerol (v/v) and 10 mg/mL DDM (detergent:protein ratio 10:1 w/w), and incubated for 10 minutes at 20°C. Solubilized material was recovered after centrifugation for 10 minutes at 120,000 x g on a TLA100.3 rotor (Beckman Coulter TL100 ultracentrifuge). Supernatant was injected on a size exclusion chromatography column (Superdex 200 10/300 GL on an Akta Purifier FPLC system, GE Healthcare) equilibrated with the same buffer but now containing only 0.5 mg/mL DDM. Chromatography was performed at 4°C and at a flow rate of 0.5 mL/min. Fractions corresponding to the main peak were collected, pooled and stored at -80°C. For further details, see [32].

Steady-state ATPase activity measurements

Measurements were performed using classical enzyme coupled assay. We measured the rate of NADH oxidation in the presence of 0.1 mg/mL lactate deshydrogenase, 0.05 mg/mL pyruvate kinase, 1 mM phosphoenolpyruvate, 0.25 mM NADH (protocol was adapted from [22]) and in the additional presence of 2 mg/mL $C_{12}E_8$ to limit time-dependent inactivation of the purified SERCA1a [20,23].

Supplementary Table 1: Ca1/2 and Vmax estimates in several selected references.

Origin of **SLN and SERCA** [a]	**Systems used** [b]	**Lipids: SLN:SERCA molar ratio** [c]	**$Ca_{1/2}$ (μM)** [d]		**Vmax in presence of SLN (% of SERCA alone)**	Advantages & ***Drawbacks***	**References**
			**no SLN**	**+ SLN**			
rSLN<br>rSERCA1a	HEK-293 cells	n.d.	0.3	0.5	143	Natural membranes, no problem of misorientation<br>Post-translational modification possible<br>***Level of expression are unknown***<br>***Stoichiometry is not controlled***	33
rSLN<br>rSERCA1a	HEK-293 cells	n.d.	0.2	0.33	95		34
rSLN<br>rSERCA2a	HEK-293 cells	n.d.	0.13	0.37	90		
rSLN<br>rat SERCA1a	Slow-twitch myocytes	n.d.	0.4	0.4	69	Native extract from rat soleus muscle<br>***Level of expression and stoichiometry are unknown***	35
hSLN<br>rSERCA1a	DOPC-DOPE bilayers	5000:15:1	0.62	0.91	100	Reconstitution in proteoliposomes<br>Control of the stoichiometry<br>***Mild detergent do not eliminate endogenous SLN***<br>***Bacteria expressed SLN with no PTM***	36
hSLN<br>DOC-extracted<br>rSERCA1a	EYPC-EYPA bilayers	140:10:1	0.4	0.6	80	Reconstitution in proteoliposomes<br>Control of the stoichiometry<br>***Mild detergent do not eliminate endogenous SLN***<br>***Bacteria expressed SLN with no PTM***	37
hSLN<br>rSERCA1a	DPC micelles	50:1 [e]	0.25	0.5	100	Control of stoichiometry<br>***Mild detergent do not eliminate endogenous SLN***<br>***Detergent may interfere with some steps of the reaction cycle***	27
rSLN<br>rSERCA1a	DOPC bilayers	160:10:1	0.2	0.5	80	Reconstitution in proteoliposomes<br>Control of the stoichiometry<br>***Low lipid/protein ratio favors oligomerisation***<br>***Mild detergent do not eliminate endogenous SLN***<br>***Synthetic peptide with no PTM***	38
hSLN<br>DOC-extracted<br>rSERCA1a	EYPC-EYPA bilayers	120:4.5:1	0.4	0.8	70	Reconstitution in proteoliposomes<br>Control of the stoichiometry<br>***Low lipid/protein ratio favors oligomerisation***<br>***Mild detergent do not eliminate endogenous SLN***	39
rSLN<br>rSERCA1a	EYPC-EYPE-EYPA bilayers	2:1<br>5:1	0.43	0.73<br>0.68	100<br>75	Reconstitution in proteoliposomes<br>Control of the stoichiometry<br>***Low lipid/protein ratio favors oligomerisation***<br>***Mild detergent do not eliminate endogenous SLN***	40
rSLN<br>rSERCA1a	DDM micelles	10:1 [f]<br>(1:1 to 50:1 tested)	0.33	0.58	100	Absence of endogenous SLN<br>Control of stoichiometry<br>High detergent/protein ratio favors monomers<br>***Detergent may interfere with some steps of the reaction cycle***	*present study*

[a] "r" referred to rabbit isoforms, "h" to human isoform.
[b] HEK-293 cells and myocytes were used for co-expression and microsomes preparation. DOPC or EYPC-EYPA bilayers obtained from co-reconstitution of native SR with recombinant or synthetic SLN.
[c] molar ratio does not take into account the possible presence of endogenous SLN. "n.d." for not determined. As a comparison, Lipids:SLN:SERCA1a ratio is about 70:1:1 to 70:2:1 mole:mole:mole in native SR.
[d] $Ca_{1/2}$ corresponds to the amount of calcium necessary to attain half-maximal activity (calcium uptake or turn-over depending on the system used).
[e] Aggregation number for DPC is 54. Proteins are distributed in about 6 mM DPC micelles (1000 micelles:50 SLN:1 SERCAa mole:mole:mole).
[f] Aggregation number for DDM is 80-120. Proteins are distributed in about 7-11 μM DDM micelles (130 micelles:10 SLN:1 SERCA1a mole:mole:mole; see Methods section for a detailed calculation).

Supplementary Figures and legends

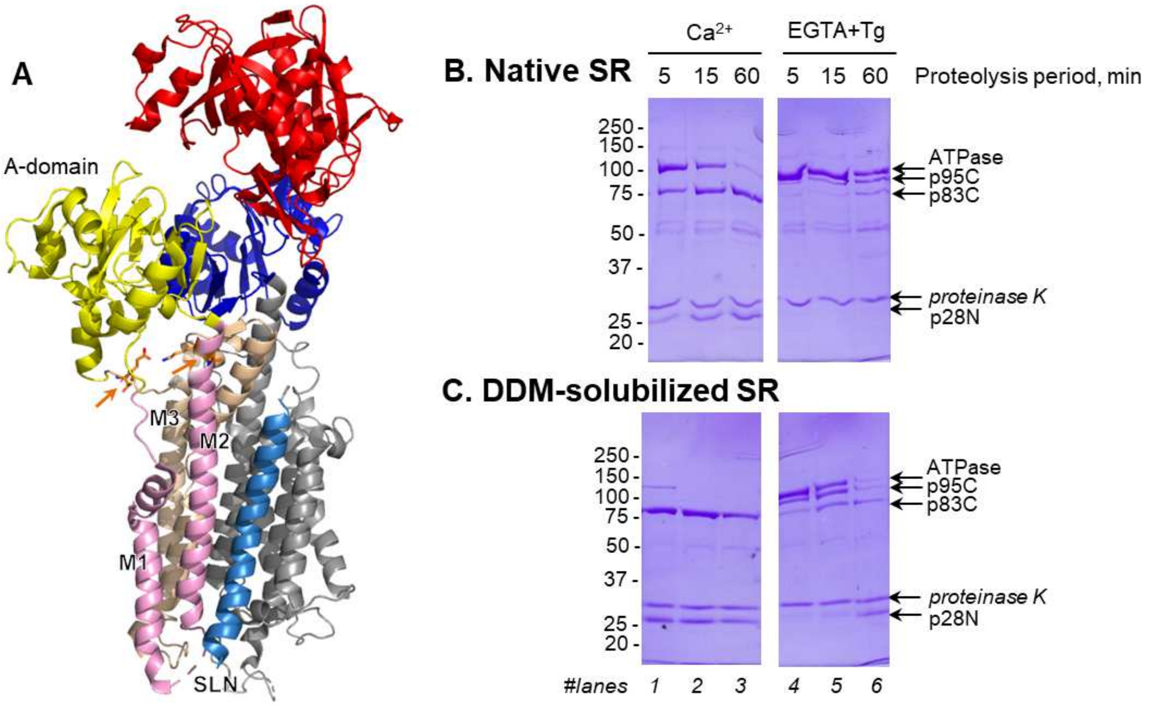

Supplementary Figure 1. Effect of DDM on the rate of cleavage of SR membrane by proteinase K. Proteinase K proteolysis was done in the presence of either 1 mM calcium (lanes 1-3) (cleavage at T242-E243) or EGTA and Thapsigargin (Tg) (lanes 4-6) (cleavage at L119-K120) for 5 to 60 minutes before loading on SDS-PAGE as mentioned in the Methods section. **A.** Topological location of proteinase K cleavage sites on SERCA1a, based on the structure of the SERCA1a/SLN complex (PDB #4H1W [41]). Structure is coloured as followed: N-domain (red), P-domain (blue), A-domain (yellow), M1-M2 (pink), M3-M4 (wheat), M5-M10 (gray) and SLN (skyblue). Residues L119-K120 and T242-E243 appear as sticks in orange and are indicated by arrows. Mg^{2+}, K^{+}, AMPPCP and water molecules present in the coordinate files are hidden for sake of clarity. **B.** Treatment by proteinase K of native membrane embedded SERCA1a from SR vesicles. **C.** Treatment by proteinase K of DDM-solubilized and SEC purified native SERCA1a. p14N fragment resulting from cleavage at L119-K120 is not visible on this SDS-PAGE (for an example see [42]).

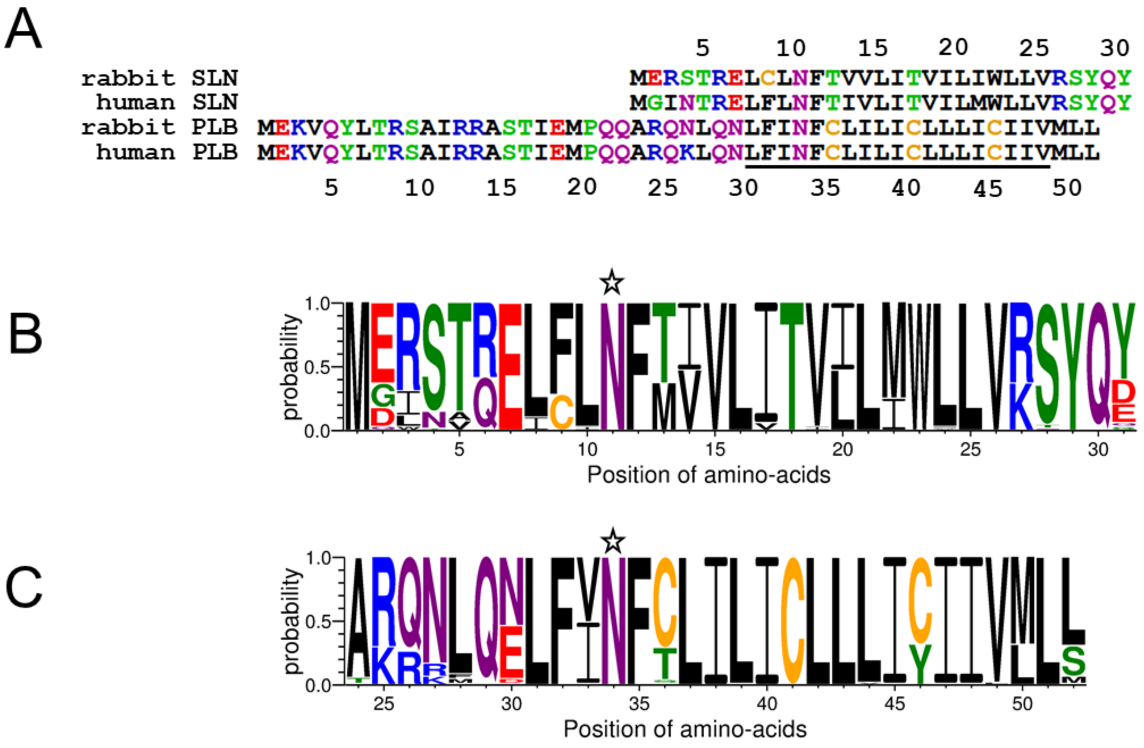

Supplementary Figure 2. Sequence alignment and sequence conservation for sarcolipin and phospholamban. Color code for the three panels is the following: acidic residues in red, basic residues in blue, cysteine in orange, Q and N in purple, other polar residues and glycine in green, and hydrophobic residues in black. **A.** We used the SEAVIEW program to align the selected sequences [43]. Upper and lower numbering referred to SLN and PLB, respectively. The region of the peptides predicted to match with the hydrophobic part of the lipid bilayer is underlined. **B.** A sequence logo was obtained from the alignment of all non-redundant SLN sequences given in UniProt (https://www.uniprot.org), i.e. 136 sarcolipin sequences covering 70 species (for detailed procedure, see [44]). Weblogo program is available at http://weblogo.threeplusone.com/ [45,46]. The height of the letters corresponds to the relative frequency of each amino acid at a given position. **C.** Sequence logo covering positions 24 to 52 of PLB sequences obtained from the alignment of 108 non-redundant PLB sequences covering 64 species. N11 for SLN and N34 for PLB (indicated by *stars*) are fully conserved among all the available sequences and appear to be critical in the inhibitory mechanism for PLB [47] and SLN [33] *via* interaction with the TM6 domain from SERCA1a (see also Supplementary Figure 10 in [41]).

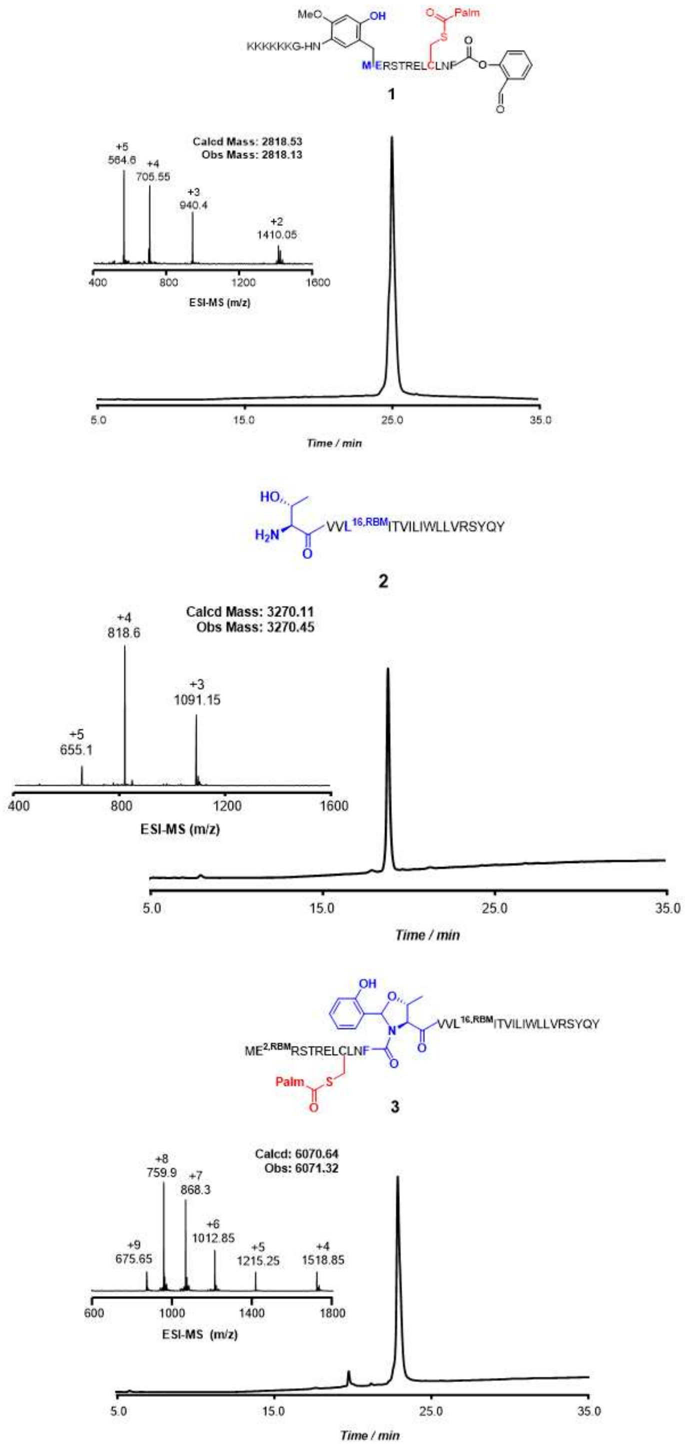

Supplementary Figure 3. RP-HPLC profiles and ESI-MS analysis of the purified peptides SLN(1-12,E^{2,RBM},C^{9,Palm})-SAL (1, top panel), SLN(13-31,L^{16,RBM}) (2, middle panel) and of the purified ligation product (3, bottom panel). Peptides were eluted from a C4 column (4.6×250 mm) using a gradient of 30% buffer B (0.1% TFA in CH_3CN) in buffer A (0.1% TFA in water) to 90% B in A over 30 min at 50°C.

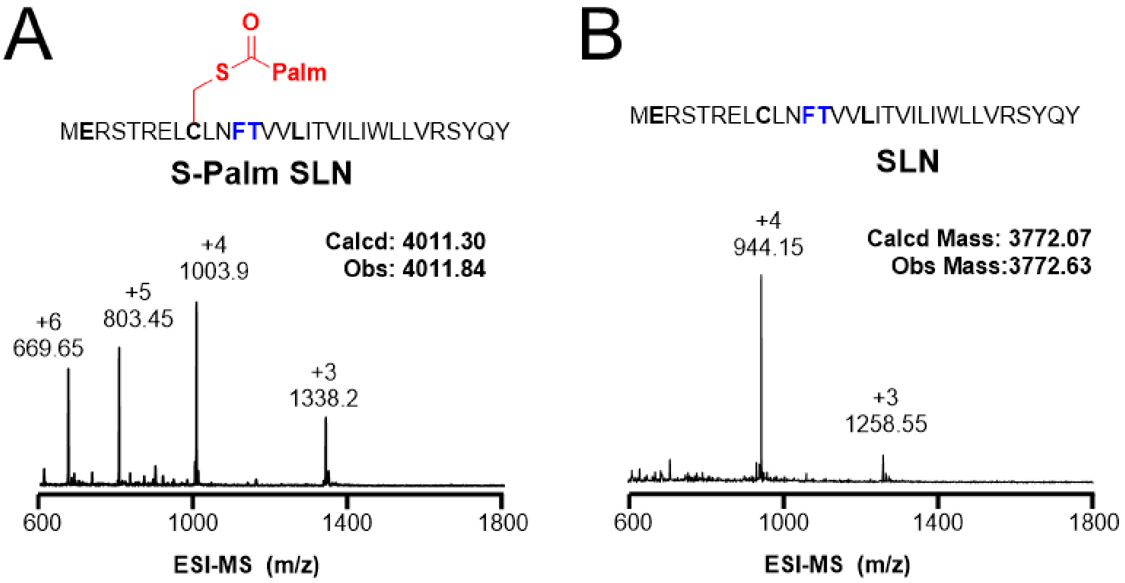

Supplementary Figure 4. Detailed ESI-MS spectra of palmitoylated SLN (Palm-SLN, A) and unacylated SLN (B).

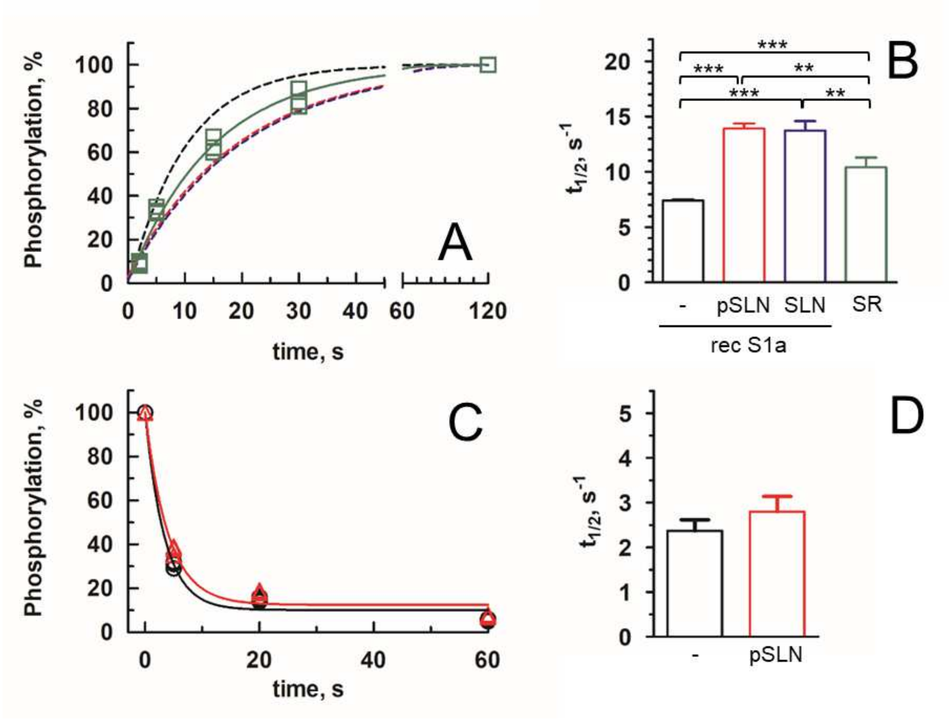

Supplementary Figure 5. Panels A & B. Rate of phosphorylation of the DDM-solubilized SR compared to that of recombinant purified SERCA1a in absence or presence of Palm-SLN or SLN. A. Kinetics of phosphorylation as presented in Figure 4C. Black, red and blue dashed lines are copies of the fit showed in Figure 4C for recS1a, recS1a+Palm-SLN and recS1a+SLN, respectively. For SR, the maximum level of phosphorylated intermediate corresponds to 6.39+/-0.20 nmoles EP/mg ATPase in agreement with the fact that only 60-80% of recombinant SERCA1a is usually active after purification [19]. **B.** Rates of phosphorylation as deduced from panel A. For SR, $t_{1/2}$=10.4 s^{-1}, with $p<0.0001$ for comparison with recS1a alone and $p=0.002$ for comparison with recS1a+Palm-SLN or recS1a+SLN. **C & D. Presence of SLN does not affect the rate of dephosphorylation. C.** Kinetic of dephosphorylation. recS1a enzyme alone (black circle) or supplemented with Palm-SLN (red triangles; pSLN:SERCA1a, 10:1, mol:mol). The data were fitted following a one phase decay. **D**. Rate of dephosphorylation as deduced from panel E. $t_{1/2}$=2.4 +/- 0.4 and 2.8 +/- 0.6 s^{-1}, $p=0.064$ indicating that rate of dephosphorylation are not significantly different. For panel B and D, data are the average of three replicates from two independent experiments.

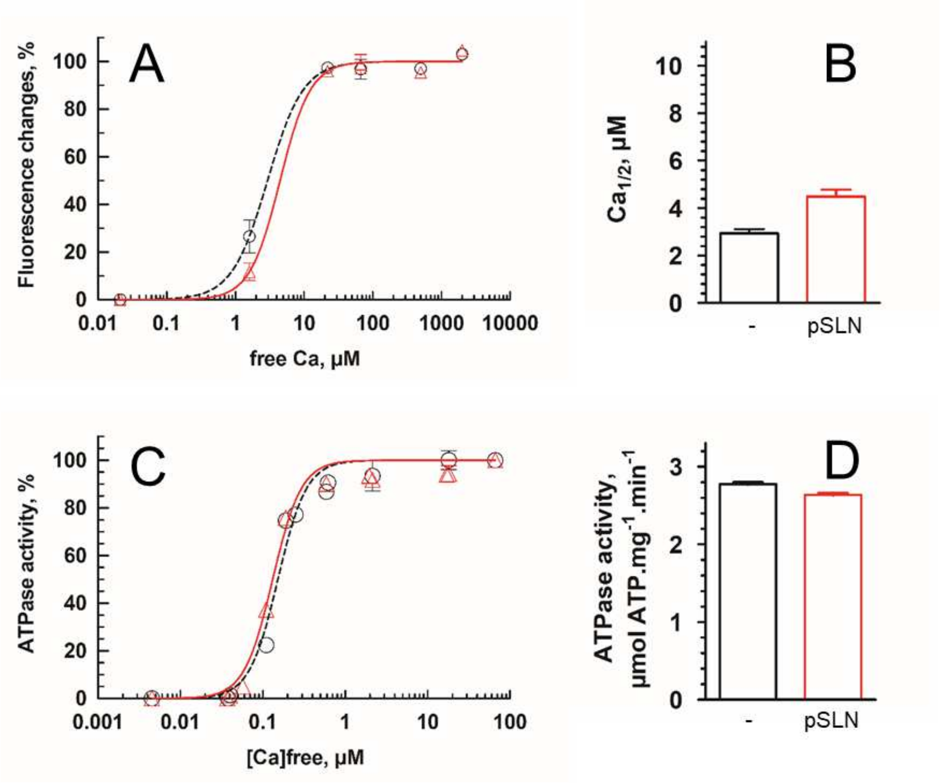

Supplementary Figure 6. Panels A & B. Affinity for calcium at equilibrium as deduced from tryptophan fluorescence changes. A. The changes in fluorescence were plotted as fractional values after normalization to 100 % of the maximal change in fluorescence in each series (recS1a, black circles; recS1a+Palm-SLN, red triangles). Each traces are the average of three independent experiments. Data were fitted with a “One site specific binding law” with Hill slope. nH are 1.67+/-0.25 and 1.96+/-0.20, respectively, thus in agreement with a cooperative binding of the two calcium ions. **B.** $Ca_{1/2}$ as deduced from panel A. $Ca_{1/2}$ are 2.94 +/- 0.30 µM and 4.48 +/- 0.52 µM, respectively, with p=0.200 indicating that the two series are not significantly different. **Panels C & D. Affinity for calcium at steady state as deduced from ATPase activity measurements. C.** ATPase activity was measured by a coupled enzyme assay for recS1a enzyme in absence (black circles) or presence of Palm-SLN (red triangles). Activities were plotted as fractional values after normalization to 100% of the maximal activity measured for each conditions. Each traces are the average of three independent experiments. Data were fitted with a one site specific binding law with Hill slope. $Ca_{1/2}$ are 0.15 +/- 0.08 µM and 0.13 +/- 0.06 µM, respectively, with p=0.248 indicating that the two series are not significantly different. **D.** Maximal turnover were 2.88 +/- 0.12 and 2.74 +/- 0.04 µmol ATP/mg/min, respectively.

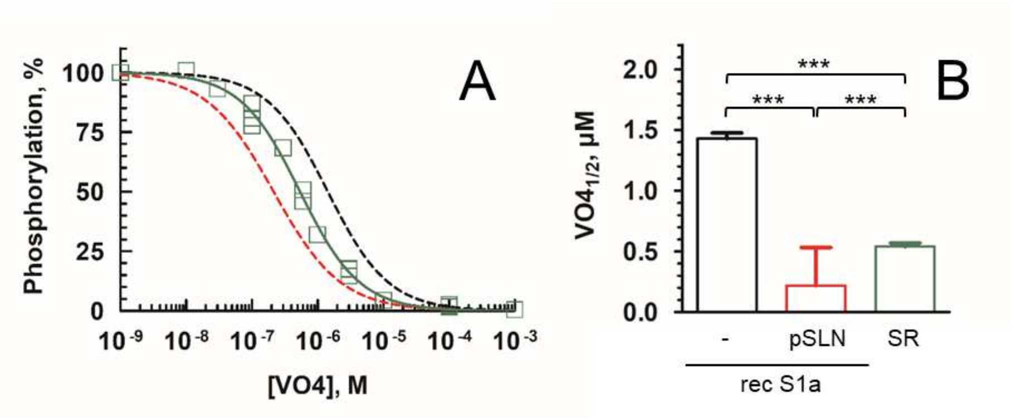

Supplementary Figure 7. Binding of orthovanadate to DDM-solubilized SR compared to that of recombinant SERCA1a in absence or presence of Palm-SLN. A. Black and red dashed lines are copies of the fit showed in Figure 5 for recS1a and recS1a+Palm-SLN, respectively. For SR, EPmax was 3.66+/-0.54 nmoles/mg of ATPase, respectively, in agreement with the fact that only 60-80% of recombinant SERCA1a is usually active after purification [19] as mentioned also in Supplementary Figure 5. All the data points are shown and are from three independent experiments. Data were fitted with a "one site specific binding law" with Hill slope. nH is 0.96+/-0.04 thus in agreement a unique binding site. **B.** Apparent affinity for vanadate as deduced from panel A. $K_{1/2}$ is 0.54+/-0.02 µM for DDM-solubilized SR while it is 1.43+/-0.08 µM and 0.22+/-0.01 µM, for recS1a and recS1a+Palm-SLN respectively, with p=0.0002 for SR vs recS1a and p<0.0001 for SR vs recS1a+pSLN.

Supplementary References

1. Amati, A. M., Graf, S., Deutschmann, S., Dolder, N. & von Ballmoos, C. Current problems and future avenues in proteoliposome research. *Biochem Soc Trans* **48**, 1473–1492 (2020).
2. Seigneuret, M. & Rigaud, J.-L. Partial separation of inwardly pumping and outwardly pumping bacteriorhodopsin reconstituted liposomes by gel filtration. *FEBS Letters* **228**, 79–84 (1988).
3. Huang, H. *et al.* Using Fluorescence Quenching Titration to Determine the Orientation of a Model Transmembrane Protein in Mimic Membranes. *Materials (Basel)* **12**, (2019).
4. de Lima Santos, H., Lopes, M. L., Maggio, B. & Ciancaglini, P. Na,K-ATPase reconstituted in liposomes: effects of lipid composition on hydrolytic activity and enzyme orientation. *Colloids Surf B Biointerfaces* **41**, 239–248 (2005).
5. Knol, J., Sjollema, K. & Poolman, B. Detergent-mediated reconstitution of membrane proteins. *Biochemistry* **37**, 16410–16415 (1998).
6. Levy, D., Gulik, A., Bluzat, A. & Rigaud, J. L. Reconstitution of the sarcoplasmic reticulum Ca(2+)-ATPase: mechanisms of membrane protein insertion into liposomes during reconstitution procedures involving the use of detergents. *Biochim Biophys Acta* **1107**, 283–98 (1992).
7. Trieber, C. A., Afara, M. & Young, H. S. Effects of Phospholamban Transmembrane Mutants on the Calcium Affinity, Maximal Activity, and Cooperativity of the Sarcoplasmic Reticulum Calcium Pump. *Biochemistry* **48**, 9287–9296 (2009).
8. Toyofuku, T., Kurzydlowski, K., Tada, M. & MacLennan, D. H. Identification of regions in the Ca(2+)-ATPase of sarcoplasmic reticulum that affect functional association with phospholamban. *J Biol Chem* **268**, 2809–2815 (1993).
9. Toyofuku, T., Kurzydlowski, K., Tada, M. & MacLennan, D. H. Amino acids Glu2 to Ile18 in the cytoplasmic domain of phospholamban are essential for functional association with the Ca(2+)-ATPase of sarcoplasmic reticulum. *J Biol Chem* **269**, 3088–3094 (1994).
10. Toyofuku, T., Kurzydlowski, K., Tada, M. & MacLennan, D. H. Amino acids Lys-Asp-Asp-Lys-Pro-Val402 in the Ca(2+)-ATPase of cardiac sarcoplasmic reticulum are critical for functional association with phospholamban. *J Biol Chem* **269**, 22929–22932 (1994).

11. Winther, A. M. *et al.* The sarcolipin-bound calcium pump stabilizes calcium sites exposed to the cytoplasm. *Nature* **495**, 265–9 (2013).

12. Toyoshima, C. *et al.* Crystal structures of the calcium pump and sarcolipin in the Mg2+-bound E1 state. *Nature* **495**, 260–264 (2013).

13. Akin, B. L., Hurley, T. D., Chen, Z. & Jones, L. R. The Structural Basis for Phospholamban Inhibition of the Calcium Pump in Sarcoplasmic Reticulum. *J. Biol. Chem.* **288**, 30181–30191 (2013).

14. Autry, J. M., Thomas, D. D. & Espinoza-Fonseca, L. M. Sarcolipin Promotes Uncoupling of the SERCA Ca2+ Pump by Inducing a Structural Rearrangement in the Energy-Transduction Domain. *Biochemistry* **55**, 6083–6086 (2016).

15. Glaves, J. P., Trieber, C. A., Ceholski, D. K., Stokes, D. L. & Young, H. S. Phosphorylation and mutation of phospholamban alter physical interactions with the sarcoplasmic reticulum calcium pump. *J Mol Biol* **405**, 707–723 (2011).

16. Aguayo-Ortiz, R. & Espinoza-Fonseca, L. M. Atomistic Structure and Dynamics of the Ca2+-ATPase Bound to Phosphorylated Phospholamban. *Int J Mol Sci* **21**, (2020).

17. Moller, J. V. *et al.* Probing of the membrane topology of sarcoplasmic reticulum Ca2+-ATPase with sequence-specific antibodies. Evidence for plasticity of the c-terminal domain. *J Biol Chem* **272**, 29015–29032 (1997).

18. Jidenko, M. *et al.* Crystallization of a mammalian membrane protein overexpressed in *Saccharomyces cerevisiae*. *Proc Natl Acad Sci U S A* **102**, 11687–11691 (2005).

19. Jidenko, M., Lenoir, G., Fuentes, J. M., Maire, M. le & Jaxel, C. Expression in yeast and purification of a membrane protein, SERCA1a, using a biotinylated acceptor domain. *Protein Expression and Purification* **48**, 32–42 (2006).

20. Montigny, C., Arnou, B., Marchal, E. & Champeil, P. Use of glycerol-containing media to study the intrinsic fluorescence properties of detergent-solubilized native or expressed SERCA1a. *Biochemistry* **47**, 12159–12174 (2008).

21. Montigny, C., Arnou, B. & Champeil, P. Glycyl betaine is effective in slowing down the irreversible denaturation of a detergent-solubilized membrane protein, sarcoplasmic reticulum Ca2+-ATPase (SERCA1a). *Biochem. Biophys. Res. Commun.* **391**, 1067–1069 (2010).

22. Møller, J. V., Lind, K. E. & Andersen, J. P. Enzyme kinetics and substrate stabilization of detergent-solubilized and membraneous (Ca2+ + Mg2+)-activated ATPase from sarcoplasmic reticulum. *J. Biol. Chem.* **255**, 1912 (1980).

23. Lund, S. *et al.* Detergent structure and associated lipid as determinants in the stabilization of solubilized Ca2+-ATPase from sarcoplasmic reticulum. *J Biol Chem* **264**, 4907–15 (1989).

24. Marchand, A. *et al.* Crystal structure of D351A and P312A mutant forms of the mammalian sarcoplasmic reticulum Ca(2+) -ATPase reveals key events in phosphorylation and Ca(2+) release. *J Biol Chem* **283**, 14867–82 (2008).

25. Clausen, J. D. *et al.* SERCA mutant E309Q binds two Ca(2+) ions but adopts a catalytically incompetent conformation. *EMBO J.* **32**, 3231–3243 (2013).

26. Geurts, M. M. G. *et al.* The SERCA residue Glu340 mediates interdomain communication that guides Ca2+ transport. *PNAS* **117**, 31114–31122 (2020).

27. Buffy, J. J. *et al.* Defining the intramembrane binding mechanism of sarcolipin to calcium ATPase using solution NMR spectroscopy. *J Mol Biol* **358**, 420–429 (2006).

28. Huang, D.-L. *et al.* Chemical Synthesis of Native S-Palmitoylated Membrane Proteins through Removable-Backbone-Modification-Assisted Ser/Thr Ligation. *Angew. Chem. Int. Ed. Engl.* **59**, 5178–5184 (2020).

29. Saint, N., Montserret, R., Chipot, C. & Penin, F. Structural and Functional Analysis of the HCV p7 Protein. in *Hepatitis C* 125–143 (Humana Press, 2009). doi:10.1007/978-1-59745-394-3_10.

30. Montigny, C. *et al.* Overexpression of Membrane Proteins in Saccharomyces cerevisiae for Structural and Functional Studies: A Focus on the Rabbit Ca2+-ATPase Serca1a and on the Yeast Lipid “Flippase” Complex Drs2p/Cdc50p. in *Membrane Proteins Production for Structural Analysis* (ed. Mus-Veteau, I.) 133–171 (Springer New York, 2014).

31. Cardi, D. *et al.* Heterologous expression and affinity purification of eukaryotic membrane proteins in view of functional and structural studies: The example of the sarcoplasmic reticulum Ca(2+)-ATPase. *Methods Mol. Biol.* **601**, 247–267 (2010).

32. le Maire, M. *et al.* Gel chromatography and analytical ultracentrifugation to determine the extent of detergent binding and aggregation, and Stokes radius of membrane proteins using sarcoplasmic reticulum Ca2+-ATPase as an example. *Nat Protoc* **3**, 1782–1795 (2008).

33. Odermatt, A. *et al.* Sarcolipin Regulates the Activity of SERCA1, the Fast-twitch Skeletal Muscle Sarcoplasmic Reticulum Ca2+-ATPase. *J. Biol. Chem.* **273**, 12360–12369 (1998).

34. Asahi, M. *et al.* Sarcolipin regulates sarco(endo)plasmic reticulum Ca2+-ATPase (SERCA) by binding to transmembrane helices alone or in association with phospholamban. *Proc Natl Acad Sci U S A* **100**, 5040–5045 (2003).

35. Tupling, A. R., Asahi, M. & MacLennan, D. H. Sarcolipin overexpression in rat slow twitch muscle inhibits sarcoplasmic reticulum Ca2+ uptake and impairs contractile function. *J Biol Chem* **277**, 44740–44746 (2002).

36. Buck, B. *et al.* Overexpression, purification, and characterization of recombinant Ca-ATPase regulators for high-resolution solution and solid-state NMR studies. *Protein Expr Purif* **30**, 253–261 (2003).

37. Douglas, J. L., Trieber, C. A., Afara, M. & Young, H. S. Rapid, high-yield expression and purification of Ca2+-ATPase regulatory proteins for high-resolution structural studies. *Protein Expr. Purif.* **40**, 118–125 (2005).

38. Hughes, E., Clayton, J. C., Kitmitto, A., Esmann, M. & Middleton, D. A. Solid-state NMR and functional measurements indicate that the conserved tyrosine residues of sarcolipin are involved directly in the inhibition of SERCA1. *J Biol Chem* **282**, 26603–26613 (2007).

39. Gorski, P. A., Glaves, J. P., Vangheluwe, P. & Young, H. S. Sarco(endo)plasmic Reticulum Calcium ATPase (SERCA) Inhibition by Sarcolipin Is Encoded in Its Luminal Tail. *J. Biol. Chem.* **288**, 8456–8467 (2013).

40. Glaves, J. P. *et al.* Interaction of a Sarcolipin Pentamer and Monomer with the Sarcoplasmic Reticulum Calcium Pump, SERCA. *Biophysical Journal* **118**, 518–531 (2020).

41. Winther, A.-M. L. *et al.* The sarcolipin-bound calcium pump stabilizes calcium sites exposed to the cytoplasm. *Nature* **495**, 265–269 (2013).

42. Juul, B. *et al.* Do Transmembrane Segments in Proteolyzed Sarcoplasmic Reticulum Ca2+-ATPase Retain Their Functional Ca2+ Binding Properties after Removal of Cytoplasmic Fragments by Proteinase K? *J. Biol. Chem.* **270**, 20123–20134 (1995).

43. Galtier, N., Gouy, M. & Gautier, C. SEAVIEW and PHYLO_WIN: two graphic tools for sequence alignment and molecular phylogeny. *Comput. Appl. Biosci.* **12**, 543–548 (1996).

44. Montigny, C. *et al.* S-Palmitoylation and S-Oleoylation of Rabbit and Pig Sarcolipin. *J Biol Chem* (Oct 9 epub).

45. Schneider, T. D. & Stephens, R. M. Sequence logos: a new way to display consensus sequences. *Nucleic Acids Res* **18**, 6097–6100 (1990).

46. Crooks, G. E., Hon, G., Chandonia, J.-M. & Brenner, S. E. WebLogo: A Sequence Logo Generator. *Genome Res.* **14**, 1188–1190 (2004).

47. Kimura, Y., Kurzydlowski, K., Tada, M. & MacLennan, D. H. Phospholamban Inhibitory Function Is Activated by Depolymerization. *J. Biol. Chem.* **272**, 15061–15064 (1997).
